# Acoustic Emission Metrology for Real-Time TIG Welding Monitoring: Spectral Characterization and Interpretability via Explainable AI

**DOI:** 10.3390/ma19194096

**Published:** 2026-09-24

**Authors:** Nandakumar Balakrishnan, Sumesh Arangot, Binoy B. Nair, Krishnakumar Ponnusamy, Dinu Thomas Thekkuden, Jithin Edacheri Veetil

**Affiliations:** 1 Department of Mechanical Engineering, Amrita School of Engineering, Amrita Vishwa Vidyapeetham, Coimbatore 641112, India; b_nandakumar@cb.students.amrita.edu (N.B.); p_kkumar@cb.amrita.edu (K.P.); 2 Department of Electronics and Communication Engineering, Amrita School of Engineering, Amrita Vishwa Vidyapeetham, Coimbatore 641112, India; b_binoy@cb.amrita.edu; 3 Mechanical and Industrial Engineering Department, Abu Dhabi University, Abu Dhabi 59911, United Arab Emirates; dinu.thekkuden@adu.ac.ae; 4 Amrita School of Artificial Intelligence, Amrita Vishwa Vidyapeetham, Coimbatore 641112, India; ev_jithin@cb.amrita.edu

**Keywords:** acoustic emission metrology, TIG welding of AA5083, frequency-domain analysis, Explainable AI (XAI), real-time edge deployment, artificial neural networks, signal non-stationarity

## Abstract

Tungsten inert gas (TIG) welding of AA5083 aluminum alloy is sensitive to heat input and shielding conditions, which can lead to defects such as porosity and burn-through that compromise weld quality. Conventional non-destructive testing methods are generally performed after welding and therefore provide limited capability for real-time process monitoring. This study presents an acoustic emission (AE)-based frequency-domain framework for real-time classification of weld conditions during TIG welding of AA5083. Acoustic signals were acquired at a sampling rate of 10 kHz and transformed into frequency-domain representations using the Fast Fourier Transform (FFT). Decision Tree (DT), Support Vector Machine (SVM), Artificial Neural Network (ANN), and ensemble-learning classifiers were evaluated using the resulting spectral features. To address the high dimensionality of the FFT representation, Principal Component Analysis (PCA) was incorporated into the modeling workflow, with the retained components determined from the model-development data. Five-fold cross-validation and grid-search-based hyperparameter tuning were used during model development, while an independent test set was reserved for final evaluation. The best-performing classifiers achieved a classification accuracy of approximately 0.99 for distinguishing good-weld, porosity, and burn-through conditions. Weld-condition labels were independently validated using visual inspection and radiographic testing. FFT and Short-Time Fourier Transform (STFT) analyses were used to characterize global and time-dependent spectral behavior, respectively, while SHapley Additive exPlanations (SHAP) were employed to identify frequency regions contributing to model predictions. A comparative analysis with time-domain statistical features further demonstrated the stronger discriminative capability of the FFT-derived representation under the investigated conditions. The proposed framework combines high classification performance with interpretable frequency-domain information and provides a basis for in-process weld-condition monitoring and quality control of TIG-welded AA5083.

## 1. Introduction

AA5083 is an Al–Mg alloy widely used in marine, transportation, and structural applications because of its favorable strength and corrosion resistance. However, achieving consistent weld quality remains challenging because aluminum alloys have high thermal conductivity, a stable surface oxide layer, and strong sensitivity to welding parameters. Variations in heat input, welding current, travel speed, electrode position, and shielding conditions can influence weld formation and may contribute to defects such as porosity, incomplete fusion, excessive penetration, and burn-through [1,2,3,4,5]. Recent investigations have demonstrated that appropriate control and optimization of TIG welding parameters are important for obtaining desirable mechanical and microstructural characteristics in AA5083 and related aluminum alloys [1,2]. These challenges highlight the need for monitoring approaches capable of identifying changes in weld quality during the welding process.

### 1.1. Critical Process Challenges in AA5083 Welding

Gas Tungsten Arc Welding (GTAW), commonly known as Tungsten Inert Gas (TIG) welding, is widely used for joining aluminum alloys because of its controllable arc characteristics and ability to produce high-quality joints. However, achieving consistent weld quality in AA5083 requires careful control of heat input, electrode position, welding current, travel speed, and shielding-gas conditions. The high thermal conductivity and strong sensitivity of aluminum alloys to thermal conditions make the welding process particularly susceptible to variations in penetration, microstructure, and defect formation. Consequently, process optimization cannot be reliably achieved through independent adjustment of individual parameters.

Heat input is one of the principal factors governing weld formation and mechanical performance. Guo et al. [2] reported a non-monotonic relationship between heat input and weld quality in TIG welding of thin AA5083 plates, identifying an optimum heat input range of approximately 296–321 J/mm, whereas excessive heat input promoted grain coarsening and deterioration of mechanical properties. Zhao et al. [1] further demonstrated that electrode height can substantially influence the tensile strength and microhardness of TIG-welded AA5083, indicating that the interaction among welding parameters must be considered when establishing a stable process window. These findings highlight the sensitivity of AA5083 weld quality to coupled process conditions rather than to a single dominant parameter.

Parameter optimization has consequently received considerable attention. Baghel [6] demonstrated the applicability of teaching–learning-based optimization and the cuckoo-search algorithm for optimizing pulsed GTAW parameters for AA5083-O and AA6061-T651. Gupta et al. [7] similarly investigated TIG welding parameters for aluminum alloys and identified parameter combinations capable of producing defect-free joints with improved mechanical performance. These studies indicate that systematic optimization can reduce the dependence on trial-and-error welding procedures.

The welding of AA5083 to dissimilar aluminum alloys introduces additional complexity because differences in composition, thermal behavior, and mechanical properties influence solidification and joint performance. Mrudula et al. [8] compared welding approaches for AA5083/AA7075 joints and demonstrated the importance of process selection in achieving suitable mechanical and corrosion properties. Methong and Komen [9] reported that a GMAW–GTAW hybrid process improved weld quality and substantially reduced porosity in aluminum-alloy joints. Arora [10] further demonstrated that post-processing of TIG-welded aluminum joints through friction-stir processing could improve strength and hardness by modifying the weld microstructure.

Defect formation remains a critical challenge. Bodaghi et al. [11] showed that welding current and travel speed influence solidification-cracking susceptibility through changes in weld-pool solidification behavior. Soysal [3] likewise highlighted the continuing susceptibility of 5xxx-series aluminum alloys to solidification cracking under unfavorable welding conditions. In addition, Liao et al. [12] demonstrated that weld-related softening and hardness gradients can influence fatigue behavior in dissimilar aluminum TIG joints. Thus, reliable AA5083 welding requires simultaneous control of process parameters, defect formation, microstructural evolution, and joint integrity.

These challenges establish the need for monitoring methods capable of identifying process-condition changes during welding, rather than relying exclusively on post-weld inspection. This requirement provides the basis for considering acoustic emission as an in-process sensing modality in the following section.

### 1.2. Role of Acoustic Emission in Weld-Quality Prediction

Acoustic emission (AE) and acoustic signal monitoring provide a non-contact means of characterizing dynamic changes during welding and have attracted increasing attention for in-process weld-quality assessment. The acoustic response generated during welding reflects transient phenomena associated with arc behavior, molten-pool dynamics, material transfer, and defect formation. Consequently, AE-based measurements can provide process-sensitive information without interrupting the welding operation. Previous investigations have demonstrated the feasibility of acoustic monitoring for detecting welding defects and process variations. Gautham et al. [13] established a time-domain acoustic-monitoring framework for TIG welding of AA5083 and demonstrated that statistical acoustic descriptors could distinguish among good-weld, porosity, and burn-through conditions. He et al. [5] investigated AE-based quality detection in laser welding, while Samimi et al. [14] demonstrated the potential of combining acoustic measurements with complementary inspection techniques for real-time observation of manufacturing processes. Liu et al. [15] further demonstrated that AE characteristics can provide information related to fatigue crack evolution in welded structures.

The application of AE to welding-quality assessment has also been extended to aluminium alloys and different welding configurations. Kannaiyan et al. [16] investigated AE monitoring during friction-stir welding of dissimilar AA5083/AA6061-T6 joints, demonstrating the sensitivity of acoustic characteristics to process conditions. Zeng et al. [17] compared deep learning and conventional machine learning approaches for AE monitoring during aluminum-alloy pulsed laser welding. Sumesh et al. [18] applied machine learning algorithms to acoustic signatures for weld-quality monitoring, establishing an early basis for data-driven interpretation of welding acoustic signals. Related acoustic-monitoring studies have also examined temporal resolution, process-emission characteristics, and burn-through detection in welding processes [19,20,21].

Recent research has expanded AE-based monitoring toward automated damage-state identification using machine learning. Tian et al. [22] investigated failure prediction in steel–ultra-high-performance-concrete (UHPC) composite beams using multi-parameter AE measurements and showed that ringing counts, energy, and frequency varied systematically with damage progression; a bidirectional gated recurrent unit model was subsequently developed for damage prediction. Wang et al. [23] combined AE monitoring with gradient-boosting machine learning models for bolt-loosening identification and demonstrated that feature selection, frequency-sensitive characteristics, dataset size, and class imbalance influence classification performance. Chai et al. [24] integrated multivariate AE parameters with machine learning classifiers to distinguish tensile damage stages in high-strength CrMoV steel, demonstrating relationships between AE characteristics, deformation stages, and crack-growth behavior. These studies collectively demonstrate that AE signals contain discriminative information that can be exploited by data-driven models for automated condition or damage identification.

However, most of these investigations have focused on structural damage, material degradation, or joint-condition assessment rather than weld-defect classification in AA5083 TIG welding. Moreover, although time- and frequency-dependent AE characteristics have both been shown to contain useful information, the use of a full-spectrum frequency-domain representation for classifying distinct TIG weld conditions remains comparatively limited. The present study therefore extends the time-domain AA5083 baseline established by Gautham et al. [13] by transforming the acoustic signals using the FFT and evaluating the resulting frequency–magnitude representation for the classification of good-weld, porosity, and burn-through conditions.

### 1.3. Machine Learning Approaches in Welding

ML provides a data-driven framework for mapping complex process monitoring signals to weld-quality conditions. In welding applications, supervised learning methods have been used for defect detection, penetration-state recognition, process monitoring, and weld-quality prediction. Sowmya et al. [25] demonstrated the importance of multi-level information fusion for machine learning-based condition diagnosis under varying operating conditions, highlighting the role of appropriate feature representation in data-driven monitoring. Xu et al. [26] investigated real-time weld-porosity prediction using arc-spectrum information and a hybrid deep learning architecture, while Xue et al. [27] developed a deep transfer learning framework for monitoring weld penetration in aluminum-alloy GTAW. These studies demonstrate that the effectiveness of ML-based monitoring depends strongly on the representation and quality of the input data.

Different model families have been investigated to balance predictive performance, interpretability, and computational complexity. Hong et al. [28] investigated deep learning-based penetration monitoring using multivariate process information, whereas Yu et al. [29] applied transfer learning to defect detection in narrow-gap GTAW. Bansal et al. [30] demonstrated the use of deep learning for radiographic detection of TIG welding defects in aluminum alloys. Wu et al. [31] investigated penetration recognition using time- and spectrum-based arc-sound information, while Luo et al. [32] developed a CNN–LSTM framework using time–frequency acoustic emission characteristics for laser-welding penetration monitoring.

For acoustic and process-signature-based monitoring, simpler machine learning approaches remain relevant because of their comparatively lower computational requirements and interpretability. Rameshkumar et al. [33] evaluated machine learning models for weld-quality monitoring using arc-signature features, demonstrating the applicability of supervised classifiers to welding-process signals. Similarly, Krishnakumar et al. [34] demonstrated the effectiveness of machine learning for acoustic emission-based tool-condition classification in precision machining, supporting the broader use of AE features for manufacturing-process-condition assessment. Sumesh et al. [35] further demonstrated the application of image-based machine learning to manufacturing-condition monitoring, illustrating the broader potential of data-driven models for automated state classification.

Collectively, these studies indicate that classifier performance cannot be considered independently of the underlying feature representation. In particular, acoustic signals are inherently high-dimensional and may contain information distributed across different temporal and spectral components. The present study therefore evaluates DT, SVM, ensemble, and ANN classifiers using the same FFT-derived acoustic representation. This controlled comparison allows the influence of the frequency-domain representation to be evaluated without confounding it with changes in the sensing modality or experimental condition.

### 1.4. Spectral Characterization and Frequency-Domain Analysis of Weld Signatures

Frequency-domain analysis provides a complementary representation of acoustic signals by resolving the distribution of signal components across frequency. Unlike time-domain statistical descriptors, which summarize global amplitude and distribution characteristics, spectral representations can reveal frequency-dependent variations associated with arc behavior, molten-pool dynamics, and defect-related transient phenomena. Jang et al. [36] investigated machine learning-based weld-porosity detection using frequency analysis of arc sound during pulsed GTAW, demonstrating the diagnostic value of spectral information for weld-defect classification. Ren et al. [37] developed a convolutional neural network using time–frequency spectrograms of arc sound for GTAW penetration classification, demonstrating the potential of preserving both spectral and temporal information for weld-state recognition.

The FFT provides an efficient means of transforming a time-domain acoustic waveform into its frequency-domain representation. While time–frequency methods can preserve temporal evolution, a global FFT provides a comparatively compact representation of the spectral content over the analyzed signal interval. The present study therefore deliberately focuses on the full-spectrum FFT frequency–magnitude representation rather than combining multiple signal domains. This design isolates the contribution of spectral information and enables the subsequent machine learning models and SHAP analysis to identify frequency regions associated with specific weld conditions. The approach provides a direct basis for evaluating whether full-spectrum acoustic information can distinguish among good-weld, porosity, and burn-through conditions in TIG-welded AA5083.

The principal research gap addressed in this work is therefore the limited direct evaluation of full-spectrum frequency-domain acoustic information against the established time-domain approach for TIG welding of AA5083 under comparable experimental and classification conditions. The present work extends the authors’ previous time-domain investigation [13] by evaluating whether FFT-based acoustic representations can improve weld-condition classification while providing interpretable spectral information. This study consequently considers the following research questions:

**RQ1:**  *What relationship exists between the frequency-domain characteristics of acoustic signals acquired during TIG welding of AA5083 and the resulting weld conditions?*

**RQ2:**  *Which machine learning model provides the best weld-quality classification performance when frequency-domain acoustic data are used?*

**RQ3:**  *Can FFT-based acoustic monitoring provide an accurate and computationally practical basis for real-time weld-quality assessment?*

## 2. Materials and Methodology

A frequency-domain investigation of the acoustic signals obtained during the TIG welding of AA 5083 was performed to evaluate the frequency-domain characteristics. The investigations, therefore, follow the same experimental setup that has been previously reported in the time-domain investigation by [13]. In addition, the same data-acquisition system and signal-recording procedure were employed throughout all of the experiments. Because the methodology was consistently applied, the time-domain and frequency-domain analyses are directly comparable.

### 2.1. Materials

AA5083 aluminum plates with dimensions of 300 mm × 150 mm × 5 mm were used as the base material for the TIG welding experiments, as illustrated in Figure 1. ER5183 aluminum–magnesium filler wire was used as the filler material because of its compatibility with the AA5083 base alloy and its suitability for TIG welding.

The chemical composition of the AA5083 base material was experimentally verified by spectrochemical analysis using optical emission spectroscopy(Optical Emission Spectrometer GNR, Milan, Italy) in accordance with ASTM E1251:2025 [38]. The measured elemental composition is presented in Table 1. The results confirm that the tested material conforms to the specified AA5083 alloy composition for the elements evaluated. The original laboratory spectrochemical test report used to verify the chemical composition is provided in Appendix A1.

The relevant physical properties of AA5083, including hardness, tensile strength, yield strength, melting point, density, and phase, were obtained from the ASM reference [39]. The plate surfaces were polished and cleaned with acetone to remove surface oxides and contaminants. A square-groove joint configuration was subsequently prepared, and the plates were accurately aligned and rigidly clamped to maintain consistent joint fit-up and minimize distortion during welding. The chemical composition of the ER5183 filler wire is presented in Table 2.

### 2.2. Experimental Methodology

The experimental methodology comprised a sequential workflow of acoustic signal acquisition, preprocessing, frequency-domain transformation, feature construction, dimensionality reduction, machine learning classification, and performance evaluation. The overall workflow is illustrated schematically in Figure 2. Acoustic signals generated during TIG welding were first acquired under controlled experimental conditions. The recorded signals were subsequently preprocessed and segmented to obtain consistent analysis intervals. The segmented signals were transformed from the time domain into the frequency domain using the FFT. The resulting frequency and magnitude values constituted the frequency-domain representation used for subsequent analyses.

The experimental dataset comprised acoustic measurements obtained from three experimentally established weld states: good weld, porosity, and burn-through. The welding parameter combinations used to establish these states are described in Section 2.5. Following signal processing, each sample was assigned the corresponding experimentally validated weld-condition label. The resulting frequency-domain dataset was subjected to dimensionality reduction using PCA before machine learning classification. DT, SVM, ANN, and ensemble-learning classifiers were subsequently evaluated. Model development incorporated five-fold cross-validation and grid-search-based hyperparameter optimization, while an independent test subset was retained for final performance evaluation. The analytical workflow was designed to evaluate whether frequency-domain acoustic information could provide reliable discrimination among the experimentally established weld states and to identify spectral characteristics associated with different welding conditions.

### 2.3. Experimental Setup

The experimental setup consisted of a TIG welding system, as shown in Figure 3, an automated welding arrangement, an acoustic sensor, and a data-acquisition system. The welding and acoustic-acquisition configuration was maintained consistently throughout the experimental trials to minimize variations unrelated to the investigated welding conditions. A ½-inch prepolarized condenser microphone was positioned 100 mm from the weld zone to capture the acoustic emissions generated during welding. The microphone output was connected to a KISTLER data-acquisition system for signal recording. A rigid positioning arrangement was used to maintain the sensor position consistently between trials. 

The welding experiments were conducted using the parameter combinations specified in Table 3. These combinations were selected during preliminary trials to establish the experimentally investigated weld states. The resulting joints were subsequently examined to verify the corresponding weld-condition labels.

### 2.4. Experimental Design

#### Weld Conditions and Parameter Selection

The welding conditions were established to obtain three experimentally distinguishable weld states: good weld, porosity, and burn-through. The same AA5083 base material, joint configuration, welding system, filler material, and acoustic-acquisition arrangement were maintained throughout the experiments. The initial process-parameter range was selected based on the baseline experimental study reported in [13] and was subsequently refined through preliminary welding trials to obtain reproducible weld conditions. The welding conditions were established using combinations of welding current, travel speed, wire-feed speed, shielding-gas flow rate, arc gap, and root gap. The complete parameter combinations used for the three weld conditions are provided in Table 3.

Reporting the complete parameter sets makes the experimental procedure self-contained and enables replication without requiring access to the previous study. The good-weld condition was established using the parameter combination corresponding to stable and uniform bead formation. The porosity condition was obtained using a parameter combination that produced internal gas-related discontinuities, whereas the burn-through condition was generated under a higher heat input condition associated with excessive penetration through the plate thickness. The parameter combinations were selected based on the preliminary experimental trials rather than attributing defect formation to a single process variable.

The resulting welds were validated independently of the acoustic measurements using visual inspection and radiographic testing (RT), where applicable. The good-weld condition was characterized by a uniform weld bead without visible defects, while RT was used to verify the absence of detectable internal discontinuities. The porosity condition was identified by internal discontinuities consistent with porosity and was further examined using RT.

The burn-through condition was identified by excessive penetration through the plate thickness. Representative photographs of the welded joints and corresponding RT images are presented in Figure 4. These inspection results were used to establish reference weld-condition labels for subsequent acoustic signal analysis.

### 2.5. Data Acquisition and Preprocessing and Measurement Uncertainty Analysis

#### 2.5.1. Acoustic Data Acquisition and Preprocessing

Acoustic signals generated during TIG welding were acquired using a ½-inch prepolarized condenser microphone positioned 100 mm from the weld zone. The microphone output was recorded using a KISTLER data-acquisition system, Model KiNOIVA LITE, Winterthur, Switzerland, at a sampling frequency of 10 kHz. The same acquisition configuration was maintained throughout the experimental trials to ensure consistent measurement conditions. Each welding trial was recorded for 90 s. To minimize the influence of transient acoustic events associated with arc initiation and extinction, the first and last 5 s of each recording were excluded from subsequent analyses. The remaining 80 s of acoustic data were retained for analysis and divided into consecutive 5 s segments. At a sampling frequency of 10 kHz, each 5 s segment contained 50,000 samples.

The segmented signals were subsequently used for frequency-domain feature extraction and weld-condition classification. A summary of the acoustic data-acquisition conditions and dataset size is provided in Table 4.

**Table 4:** Summary of acquired acoustic data.

Signature	Sound
Sampling rate	10 kHz
Effective data duration per test case	80 s
No. of data points per test case	800,000
Total weld conditions	3 (undisturbed arc weld, porosity, burn-through)
Data points per weld condition	3,200,000
Total data points acquired	9,600,000

#### 2.5.2. Measurement Uncertainty Analysis

The reliability of the acoustic measurement system was evaluated by considering Type A and Type B uncertainty components in accordance with the principles of the Guide to the Expression of Uncertainty in Measurement (GUM). Type A uncertainty was associated with the statistical repeatability of the measurements, whereas Type B uncertainty included contributions from the measurement system and experimental configuration. The principal uncertainty components considered were statistical repeatability ($u_{stat}$), sensor calibration ($u_{cal}$), spatial positioning ($u_{pos}$), and data-acquisition quantization ($u_{quant}$).

The maximum relative standard deviation of the acoustic-amplitude features was 1.0%, while the estimated contributions from sensor calibration, spatial positioning, and DAQ quantization were 0.5%, 0.4%, and approximately 0.0015%, respectively. The combined standard uncertainty was determined using the root-sum-square formulation. [Equation (1)]

(1) $$U=\sqrt{U_{stat}^{2}+U_{cal}^{2}+U_{pos}^{2}+U_{quant}^{2}}$$

where $u_{c}$ is the combined standard uncertainty and $u_{stat}$, $u_{cal}$, $u_{pos}$, and $u_{quant}$ represent the individual standard uncertainty components. The resulting combined standard uncertainty was 1.2%.

Using a coverage factor of k=2, the expanded uncertainty was calculated as

$$U=ΚU_{c}$$

corresponding to an approximately 95% coverage probability under the assumption of a normal distribution. The resulting overall expanded uncertainty was 2.4%. This uncertainty level indicates that the acoustic measurement configuration provides adequate repeatability and measurement precision for the frequency-domain analysis and subsequent machine learning-based weld-condition classification. The principal measurement and metrological specifications are summarized in Table 5.

### 2.6. Frequency-Domain Feature Extraction and Dimensionality Reduction

The preprocessed acoustic signals were transformed from the time domain into the frequency domain using the FFT. Each acoustic segment had a duration of 5 s and was sampled at 10 kHz. The FFT was applied to each segment to obtain its frequency-domain representation, and the magnitude of each frequency component was subsequently calculated. This representation was used to characterize the spectral response associated with the different weld conditions.

For a discrete acoustic signal $x\left[n\right]$ containing N samples, the Fourier Transform was calculated as [Equation (2)]

(2) $$X\left(k\right)=\sum_{n=0}^{N-1}x\left(n\right)e^{-\frac{j2\pi kn}{N}},\;\;\;k=0,1,\ldots ,N-1$$

where $x\left[n\right]$ represents the discrete acoustic signal, $X\left[k\right]$ denotes the complex Fourier coefficient corresponding to frequency-bin index k, N is the number of samples in the signal segment, and $j=\sqrt{-1}$.

The magnitude of each frequency component was calculated as [Equation (3)]

(3) ∣Xk∣=(Re(X[k]))2+(Im(X[k]))2

where $|X\left[k\right]|$ is the magnitude of the kth frequency component, $Re\left(X\left[k\right]\right)$ represents the real part of the FFT output, $Im\left(X\left[k\right]\right)$ represents the imaginary part of the FFT output, and k denotes the frequency-bin index. In the spectral plots, the FFT magnitude is presented in arbitrary units (a.u.), since the analysis uses the numerical magnitude of the recorded acoustic signal rather than a separately calibrated sound-pressure level.

Because the acoustic signals were sampled at 10 kHz, the Nyquist frequency was 5 kHz. Accordingly, the physically interpretable frequency range extended from 0 to 5 kHz. Frequency components above 4.9 kHz were excluded because they contained negligible energy under the experimental conditions. No interpolation or smoothing was applied so that the measured spectral characteristics were preserved. The resulting FFT representation formed the high-dimensional frequency-domain input used for subsequent dimensionality reduction. The frequency-domain spectra of the three weld conditions are presented in Figure 5. The initial FFT representation contained 49,800 frequency-domain features per sample. The use of such a high-dimensional representation relative to the available number of observations can result in a high predictor-to-sample ratio and increase the risk of overfitting. Therefore, PCA was incorporated as a dimensionality reduction step before machine learning classification.

The dataset contained 180 samples, with 60 samples corresponding to each weld condition. The training/validation portion comprised 70% of the dataset, corresponding to 126 samples, while the remaining 30% was reserved for independent testing. The number of principal components was therefore limited to 125, corresponding to N−1, where N=126 is the number of training/validation observations. This constraint follows from the maximum rank of the centered data matrix when the number of observations is smaller than the number of original predictors. The PCA transformation was used to project the original high-dimensional FFT feature space onto the first 125 principal components, thereby reducing the dimensionality of the input representation while retaining the dominant variance directions in the training/validation data. The resulting reduced feature representation was subsequently supplied to the machine learning classifiers for weld-condition classification.

### 2.7. Machine Learning Classification and Validation

The processed frequency-domain features were evaluated using four classifier families: DT, SVM, ANN, and ensemble-learning models. The dataset consisted of 180 acoustic samples representing three experimentally established weld conditions. The data were divided into 70% for model development and 30% for independent testing. The model-development subset was used for model fitting, five-fold cross-validation, and hyperparameter optimization, while the independent test set was kept separate from model selection and tuning. Following optimization, each classifier was retrained using the complete model-development subset and evaluated on the independent test set. PCA was applied to the FFT-derived high-dimensional representation using the model-development data, and the same transformation was subsequently applied to the independent test data. Classification accuracy, precision, sensitivity, specificity, F1-score, ROC-AUC, and Cohen’s kappa were used to assess predictive performance, and class-discrimination.

The Decision Tree models, the classifier structure, and associated hyperparameters were optimized during model development. The SVM classifier was evaluated using three kernel functions: linear, quadratic, and Gaussian. The linear kernel was included as a baseline to assess the performance of a linear decision boundary, while the quadratic and Gaussian kernels were evaluated to determine whether nonlinear decision boundaries could provide improved separation of the weld-condition classes. The kernel type and associated hyperparameters were selected through the model-development procedure using five-fold cross-validation and grid-search-based hyperparameter tuning. The independent test set was not used during kernel selection or hyperparameter optimization. The ANN models were evaluated using three network-width configurations designated NNN, MNN, and WNN, corresponding to single-hidden-layer architectures containing 10, 25, and 100 hidden neurons, respectively. The remaining network settings were maintained according to the optimized ANN configuration. This arrangement enabled the effect of hidden-layer width on classification performance to be assessed systematically. Several ensemble-learning configurations were also evaluated to determine whether combining multiple weak or complementary learners could improve classification robustness. The final performance of each optimized classifier was assessed using the independent test set. Classification accuracy, precision, sensitivity, specificity, F1-score, ROC-AUC, and Cohen’s kappa were used to characterize predictive performance and class discrimination.

## 3. Results and Discussion

### 3.1. Spectral and Time–Frequency Characterization of Weld Conditions

The acoustic responses associated with the three experimentally validated weld conditions were examined in both the frequency and time–frequency domains. The FFT was used to characterize the overall frequency distribution of the acoustic energy, while the STFT was used to examine the temporal evolution of the spectral content. These two representations provide complementary information for distinguishing the acoustic characteristics associated with the good-weld, porosity, and burn-through conditions.

Because the acoustic signals were acquired at a sampling frequency of 10 kHz, the maximum physically interpretable frequency is 5 kHz, according to the Nyquist criterion. Accordingly, the frequency axis of the revised FFT spectra is presented within the valid 0–5 kHz range. The previous incorrect labeling of the horizontal axis has been corrected so that the plotted frequency information corresponds to the actual frequency-domain representation.

#### 3.1.1. Good Weld (Stable Arc Regime)

The FFT magnitude spectrum for the good-weld condition is presented in Figure 6a, and the corresponding STFT spectrogram is shown in Figure 7a. The FFT spectrum exhibits a comparatively stable distribution of acoustic energy, with the dominant contribution concentrated in the lower-frequency region. No pronounced isolated broadband peaks are observed, indicating a relatively consistent acoustic response during the welding process.

The corresponding STFT spectrogram provides additional information regarding the temporal stability of the spectral response. The acoustic energy remains comparatively continuous over time, particularly within the lower-frequency region, rather than appearing as repeated high-energy transient events. This behavior is consistent with a relatively stable welding process and provides a spectral reference for comparison with the defect-containing weld conditions.

#### 3.1.2. Porosity (Transient Gas-Induced Disturbances)

The FFT magnitude spectrum corresponding to the porosity condition is presented in Figure 6b, while the associated STFT spectrogram is shown in Figure 7b. In comparison with the good-weld condition, the porosity signal exhibits more pronounced spectral activity over a broader frequency range.

The STFT representation further reveals localized, intermittent high-energy events, particularly within the approximately 2–4.5 kHz frequency range. These transient spectral features indicate that the acoustic response associated with the porosity condition is temporally non-uniform. The occurrence of localized broadband acoustic activity is consistent with transient disturbances during the welding process and provides a distinguishable time–frequency characteristic of the porosity condition.

#### 3.1.3. Burn-Through (Melt Pool Instability)

The FFT magnitude spectrum for the burn-through condition is presented in Figure 6c, and its corresponding STFT spectrogram is shown in Figure 7c. Compared with the good-weld condition, the burn-through signal exhibits stronger low-frequency spectral activity.

The STFT representation shows comparatively persistent acoustic energy in the lower-frequency region, approximately 0.1–1.5 kHz. Unlike the localized transient behavior observed in the porosity condition, the spectral activity associated with burn-through is more sustained over time. This behavior is consistent with a persistent change in the welding process associated with excessive penetration and molten-pool instability.

Overall, the FFT and STFT analyses reveal distinguishable acoustic characteristics among the three weld conditions. The good-weld condition exhibits comparatively stable spectral behavior, the porosity condition is characterized by intermittent higher-frequency activity, and the burn-through condition exhibits more persistent low-frequency spectral activity. These observations provide physical context for the frequency-domain features used subsequently for machine learning-based weld-condition classification. Table 6 summarizes the correlation between the observed spectral characteristics and the underlying physical welding mechanisms.

### 3.2. Model Interpretability and Physical Correlation of Features

The high classification performance of the machine learning models was further examined using SHapley Additive exPlanations (SHAP) version 0.44.1 to assess the contribution of the frequency-domain features to the model predictions. SHAP was used to identify the spectral regions that had the greatest influence on the classification of the three experimentally validated weld conditions. This analysis provides an interpretable basis for relating the model predictions to the acoustic characteristics observed in the spectral and time–frequency analyses.

#### SHAP Analysis of Defect Fingerprints

The SHAP feature-importance results are presented in Figure 8. The analysis shows that the classifier assigns different levels of importance to different frequency regions for the three weld conditions, indicating that the model does not rely uniformly on the complete frequency spectrum for classification. For the good-weld condition, greater feature importance is observed in the approximately 1.0–2.0 kHz region. This frequency range is consistent with the relatively stable spectral behavior observed for the good weld in the FFT and STFT analyses. The result indicates that spectral information in this region contributes to distinguishing the stable welding condition from the defect-containing conditions.

For the porosity condition, the highest feature importance is concentrated approximately within the 2.5–4.1 kHz range. This result agrees with the higher-frequency transient activity observed in the corresponding STFT representation. The agreement between the SHAP analysis and the independent spectral observations indicates that this frequency region contains information that is useful for distinguishing porosity from the other weld conditions. The observed association is physically consistent with transient disturbances occurring during porosity formation; however, the acoustic measurements do not directly resolve individual gas bubble formation or rupture events.

For the burn-through condition, greater feature importance is observed at frequencies below approximately 1.5 kHz. This finding is consistent with the persistent low-frequency acoustic activity observed in the FFT and STFT analyses. The concentration of SHAP importance in this region indicates that low-frequency spectral information contributes substantially to the identification of burn-through. The observed behavior is consistent with larger-scale variations in the welding process and molten-pool dynamics associated with excessive penetration.

Overall, the SHAP results identify condition-dependent spectral regions that are consistent with the independently observed acoustic characteristics. The porosity condition is primarily associated with higher-frequency components in the approximately 2.5–4.1 kHz range, whereas burn-through is more strongly associated with frequencies below approximately 1.5 kHz. The good-weld condition shows greater importance in the approximately 1.0–2.0 kHz region. These findings provide an interpretable connection between the frequency-domain information used by the classifier and the experimentally observed acoustic signatures of the three weld conditions.

### 3.3. Comparison of Global FFT vs. STFT

The FFT and STFT provide complementary representations of the acoustic signals generated during TIG welding. The FFT provides a global representation of the frequency content, whereas the STFT additionally describes the temporal evolution of the spectral components. In the present study, FFT-derived features were used as the input to the machine learning models, while STFT was employed to provide complementary time–frequency information for interpretation of the acoustic responses. The STFT analysis provides additional information regarding the temporal characteristics of the weld-condition signatures. In particular, the acoustic response associated with porosity exhibits intermittent higher-frequency activity, whereas burn-through is characterized by more persistent low-frequency activity. These observations complement the spectral characteristics identified from the global FFT analysis. It should be noted that the present study does not constitute a direct comparison of the classification performance of FFT- and STFT-derived feature sets, because the machine learning models were developed using FFT-derived features. Instead, the comparison focuses on the computational characteristics and analytical roles of the two representations. The feature extraction time, memory footprint, and inference latency obtained for the two approaches are summarized in Table 7.

The computational comparison indicates that the FFT-based approach requires lower processing time and memory than the STFT-based approach within the evaluated implementation. This lower computational burden is advantageous for acoustic-monitoring applications where low processing latency and limited computational resources are important. However, the results should not be interpreted as evidence that FFT is universally superior to STFT. The two methods serve different purposes within the proposed framework. FFT provides the primary frequency-domain representation used for machine learning classification, whereas STFT provides complementary time–frequency information for interpreting the temporal evolution of the acoustic response.

Overall, the results demonstrate that FFT offers a computationally efficient spectral representation for the present classification framework, while STFT contributes additional information regarding the temporal behavior of weld-condition-related acoustic features. Thus, the two approaches are considered complementary rather than competing signal representations.

### 3.4. Model Performance

The performance of the machine learning models was evaluated using the FFT-derived frequency-domain acoustic features. Four classifier families—DT, SVM, ANN, and ensemble models—were investigated to assess their capability to discriminate among the good-weld, porosity, and burn-through conditions. The models were developed and evaluated using the data-partitioning, dimensionality reduction, cross-validation, and hyperparameter-optimization procedures described in Section 2.7. The final performance of each optimized model was assessed using the independent test set.

The principal hyperparameter settings used for the evaluated classifiers are summarized in Table 8. These settings include the splitting criterion and maximum number of splits for DT, kernel configuration for SVM, network architecture and training parameters for ANN, and learner configuration for the ensemble model. The classifiers were assessed using classification accuracy, ROC-AUC, and Cohen’s Kappa, together with precision, sensitivity, specificity, F1-score, and related diagnostic measures where applicable.

#### 3.4.1. Decision Tree

The Decision Tree classifier was evaluated using different maximum-split configurations to examine the effect of tree complexity on classification performance. The results for the DT configurations investigated are summarized in Table 9. The models showed comparable performance across the tested split settings, indicating that the frequency-domain representation provided substantial class-discriminative information. The Gini Index, with a maximum of 100 splits, was selected for the detailed evaluation. The corresponding classification results are presented in Table 10. The selected DT correctly classified 59 burn-through samples, 59 good-weld samples, and 58 porosity samples. Only a small number of observations were misclassified, primarily between the porosity and burn-through classes.

**Table 9:** Decision tree model performance summary.

Decision Tree			
Model Hyperparameters	Classification Accuracy %	ROC Value	Cohen’s Kappa
Max. no of splits = 100	0.98	0.98	0.86
Max. no of splits = 20			
Max. no of splits = 4			

**Table 10:** Confusion matrix of the Decision Tree classifier trained using FFT-derived frequency-domain acoustic features.

		**B**	**G**	**P**
Actual Class	B	59	1	0
	G	0	59	0
	P	2	0	58
**Evaluated Parameters**				
Classification Accuracy	0.98		Precision	0.98
ROC Value	0.98		F1-Score	0.98
Kappa Statistics	0.97		Specificity	0.99
Sensitivity	0.98		Prevalence	0.33
False Discovery Rate (FDR)	1.65		Miss rate	1.6

(B: burn-through, G: good weld, and P: porosity).

A worked example showing how the evaluation parameters are calculated from this confusion matrix is provided in Appendix A.2. The model achieved a classification accuracy of approximately 0.98, with a ROC-AUC of approximately 0.98 and Cohen’s Kappa of approximately 0.97. The sensitivity, specificity, precision, and F1-score were also high, demonstrating that the DT provided a strong baseline for discrimination of the investigated weld conditions. The same calculation procedure was applied to the SVM, ANN, and ensemble models.

#### 3.4.2. Support Vector Machine

The SVM classifier was evaluated to determine whether alternative kernel functions could improve the separation of the weld-condition classes. In accordance with the revised model-development procedure, linear, quadratic, and Gaussian kernel configurations were considered rather than restricting the analysis to a single kernel type. The comparative SVM performance is presented in Figure 9. The Quadratic SVM provided the strongest classification performance among the evaluated configurations and was selected for detailed evaluation. The corresponding confusion matrix is presented in Table 11.

The selected SVM correctly classified the reported burn-through and good-weld samples, with only one porosity sample misclassified as burn-through. The model achieved approximately 0.99 classification accuracy, 0.99 ROC-AUC, and 0.99 Cohen’s Kappa. The results indicate that the nonlinear decision boundary provided by the quadratic kernel was effective in separating the acoustic feature distributions associated with the three weld conditions. The remaining porosity-to-burn-through error indicates that some overlap between these defect classes remains within the frequency-domain representation.

#### 3.4.3. Artificial Neural Network Model for Defect Classification

Artificial Neural Network (ANN) models were evaluated to investigate whether different network widths influence the classification of weld conditions using the FFT-derived frequency-domain acoustic features. Three single-hidden-layer ANN configurations were considered—Narrow Neural Network (NNN), Medium Neural Network (MNN), and Wide Neural Network (WNN)—containing 10, 25, and 100 neurons in the hidden layer, respectively. This terminology is used to distinguish the ANN configurations according to their hidden-layer width.

The comparative performance of the three ANN configurations is presented in Figure 10. The purpose of evaluating different network widths was to determine whether increasing the number of hidden neurons provides a measurable improvement in classification performance. The NNN configuration with 10 hidden neurons was selected for detailed evaluation because it provided competitive performance without introducing unnecessary network complexity.

The confusion matrix of the selected ANN is presented in Table 12. The model correctly classified the reported burn-through and good-weld samples, while one porosity sample was classified as burn-through. The resulting classification accuracy, ROC-AUC, and Cohen’s Kappa were approximately 0.99.

The results indicate that increasing the hidden-layer width from 10 to 25 or 100 neurons did not provide a substantial improvement in classification performance. The competitive performance of the NNN configuration therefore suggests that the frequency-domain representation already contains sufficient discriminative information for the classification task. A relatively compact ANN also reduces unnecessary model complexity, which is desirable when the available dataset is limited. The training behavior of the selected ANN was additionally examined using the convergence analysis. The convergence result provides information about the learning behavior of the network during training, while the use of cross-validation, dimensionality reduction, and hyperparameter tuning provides additional safeguards against overfitting.

#### Convergence and Overfitting Assessment

The training convergence of the selected ANN was examined using the training-loss curve, as shown in Figure 10a. The convergence curve provides an indication of how the training error evolved during network optimization and whether the learning process approached a stable solution. The corresponding confusion matrix is presented in Table 13, providing the class-level prediction results for the selected ANN. The combination of the convergence analysis and independent test-set confusion matrix provides complementary information: the convergence plot describes the training behavior of the network, whereas the confusion matrix evaluates its classification performance on the evaluated data.

To reduce the potential for overfitting associated with the high-dimensional frequency-domain representation, the revised workflow incorporated PCA-based dimensionality reduction, five-fold cross-validation, and grid-search-based hyperparameter tuning during model development. In addition, the independent test data were kept separate from hyperparameter selection. These procedures provide safeguards against selecting an ANN configuration solely based on its performance on the training data.

The comparison of NNN, MNN, and WNN further indicates that a larger number of hidden neurons did not result in a meaningful improvement in classification performance. The competitive performance of the compact NNN configuration therefore supports the use of a relatively simple ANN architecture for the present dataset.

#### 3.4.4. Ensemble Models

Several ensemble-learning approaches were evaluated, including Boosted Trees, Bagged Trees, Subspace Discriminant, Subspace K-Nearest Neighbors, and RUSBoosted Trees. The purpose was to determine whether combining learners or using different feature subspaces could improve the robustness of weld-condition classification. The comparative ensemble results are presented in Figure 11. Among the evaluated ensemble approaches, the Subspace Discriminant classifier provided the strongest overall performance and was selected for detailed evaluation. Its corresponding confusion matrix is presented in Table 14. The selected ensemble classifier correctly classified the reported burn-through and good-weld samples, with one porosity sample misclassified as burn-through. The resulting classification accuracy, ROC-AUC, and Cohen’s Kappa were approximately 0.99.

The results demonstrate that the ensemble approach can effectively exploit the discriminative information contained in the frequency-domain acoustic representation. However, the residual confusion between porosity and burn-through indicates that some similarity remains between their acoustic signatures.

#### 3.4.5. Comparison with Previous Studies

The classification performance obtained in this study is comparable with previous acoustic emission-based welding studies, while highlighting the benefit of frequency-domain representation. The FFT-derived features achieved approximately 0.98–0.99 accuracy across the evaluated classifiers, compared with approximately 0.86 for the time-domain statistical baseline. This improvement indicates that frequency-specific acoustic information provides greater discriminative capability for distinguishing good-weld, porosity, and burn-through conditions.

Unlike approaches focused primarily on post-weld inspection or time-domain descriptors, the proposed framework uses acoustic signals acquired during welding, enabling potential in-process weld-condition monitoring. In addition, SHAP analysis provides interpretable information on the frequency regions contributing to classification decisions, thereby linking model predictions with the observed spectral characteristics.

Direct numerical comparison with previous studies should be interpreted cautiously because differences in materials, defect types, sensing configurations, datasets, and evaluation protocols can affect classification performance. Nevertheless, the present results demonstrate the potential of FFT-based acoustic features combined with machine learning and interpretability analysis for in-process monitoring of TIG-welded AA5083.

### 3.5. Comparative Analysis of Time- and Frequency-Domain Approaches

A comparative study was conducted to assess whether the application of time-domain or frequency-domain features based on acoustics can be effective as a means for identifying weld defects. Comparison was done using the “best” model for each of the four categories of classifiers: Decision Trees (DTs), Support Vector Machines (SVMs), Neural Networks (NNs), and ensembles.

Classification accuracy, for both sets of feature representations, is illustrated in Figure 12. Statistical features extracted from time-domain signal data were utilized in model development for the time-domain method. Due to the large degree of overlap among weld conditions, these features had limited ability to provide discriminant information. Maximum classification accuracy achieved by the time-domain method was 0.86, which was obtained by DT models utilizing the Gini Index and Twoing criteria. ANN and ensemble methods produced slightly lower accuracies, while SVM models produced the lowest accuracy at 0.81. The above results indicate that time-domain features were unable to capture the specific characteristics of the weld defects during the welding process.

On the other hand, the frequency-domain method demonstrated a significant increase in classification accuracy for all the classifiers evaluated. Frequency–magnitude features derived from FFT enabled better separation of classes and an increase in classification accuracy to 0.98–0.99. The Artificial Neural Network configurations with a two-hidden-layer model produced the highest accuracy, 0.99, while DT, ensemble, and SVM models all demonstrated consistently high levels of accuracy. The results clearly show that frequency-domain features enabled the identification of spectral characteristics which were not present in time-domain representations.

The comparative studies demonstrated that frequency-domain acoustic features were superior to time-domain statistical features for weld-defect classification. The frequency-domain method demonstrated a higher level of accuracy, a higher level of robustness, and consistent performance with respect to the various classifiers; therefore, the frequency-domain method appears to be more appropriate for reliable weld-quality monitoring in the TIG welding of AA5083.

## 4. Conclusions

This study investigated an acoustic signal-based framework for the classification of good-weld, porosity, and burn-through conditions during TIG welding of AA5083 using frequency-domain analysis and machine learning techniques. The principal conclusions are summarized below.

• Frequency-domain acoustic characterization: FFT-based analysis revealed distinguishable spectral characteristics among the investigated weld conditions. The good-weld condition exhibited comparatively stable spectral behavior, porosity was associated with intermittent higher-frequency activity, and burn-through exhibited more persistent low-frequency acoustic activity. These observations demonstrate the potential of acoustic spectral information for in-process weld-condition monitoring.
• Decision Tree classification: The Decision Tree provided a strong and interpretable baseline for weld-condition classification, achieving approximately 0.98 classification accuracy. Its performance demonstrates that the FFT-derived representation contains sufficient information for rule-based separation of the investigated weld states.
• SVM classification: The evaluation of multiple SVM kernel configurations demonstrated that nonlinear classification can effectively exploit the frequency-domain feature representation. The selected SVM achieved approximately 0.99 classification accuracy, indicating strong separation of the three weld conditions.
• ANN classification: Different ANN widths were investigated using the NNN, MNN, and WNN configurations. The compact single-hidden-layer architecture with 10 hidden neurons provided competitive performance, achieving approximately 0.99 classification accuracy. The results indicate that increasing network width does not necessarily provide a corresponding improvement in classification performance.
• Ensemble classification: Among the evaluated ensemble approaches, the Subspace Discriminant model provided approximately 0.99 classification accuracy. Its performance further demonstrates that the frequency-domain representation remains effective across different classifier architectures.
• Model reliability and interpretability: Five-fold cross-validation, hyperparameter tuning, dimensionality reduction, and an independent test set were incorporated into the revised modeling framework to reduce overfitting and improve the reliability of performance estimates. SHAP analysis further identified condition-dependent frequency regions, contributing to model predictions and providing an interpretable connection between the learned spectral features and the observed acoustic characteristics.
• Time-domain versus frequency-domain representation: The comparative analysis showed that the selected time-domain statistical features—mean, standard deviation, maximum, minimum, variance, median, mode, skewness, kurtosis, and sum—provided lower classification performance than the FFT-derived frequency-domain representation under the investigated conditions. The frequency-domain approach therefore provided greater discriminative capability for the three weld states considered in this study.
• Practical implications for TIG welding of AA5083: The results demonstrate the feasibility of using acoustic sensing and frequency-domain machine learning as an in-process monitoring approach for identifying weld-condition changes during TIG welding. Such a system could provide an early indication of porosity or burn-through without relying exclusively on post-weld inspection. This has potential practical value for process monitoring and quality-control applications where rapid identification of abnormal welding conditions is required.
• FFT and STFT: FFT and STFT served complementary purposes in the proposed framework. FFT provided the primary frequency-domain representation used for classification, whereas STFT provided time–frequency information for interpreting transient and persistent acoustic behavior. The computational comparison indicated lower processing and memory requirements for FFT within the evaluated implementation.

Overall, the findings demonstrate that frequency-domain acoustic analysis combined with machine learning and model interpretability provides an effective framework for in-process monitoring of TIG-welded AA5083. The combination of spectral discrimination, high classification performance, and interpretable feature analysis establishes a basis for real-time weld-quality monitoring, while future studies should evaluate broader welding conditions, industrial operating environments, and combined time- and frequency-domain feature representations to assess robustness and generalization.

## Figures and Tables

**Figure 1:**
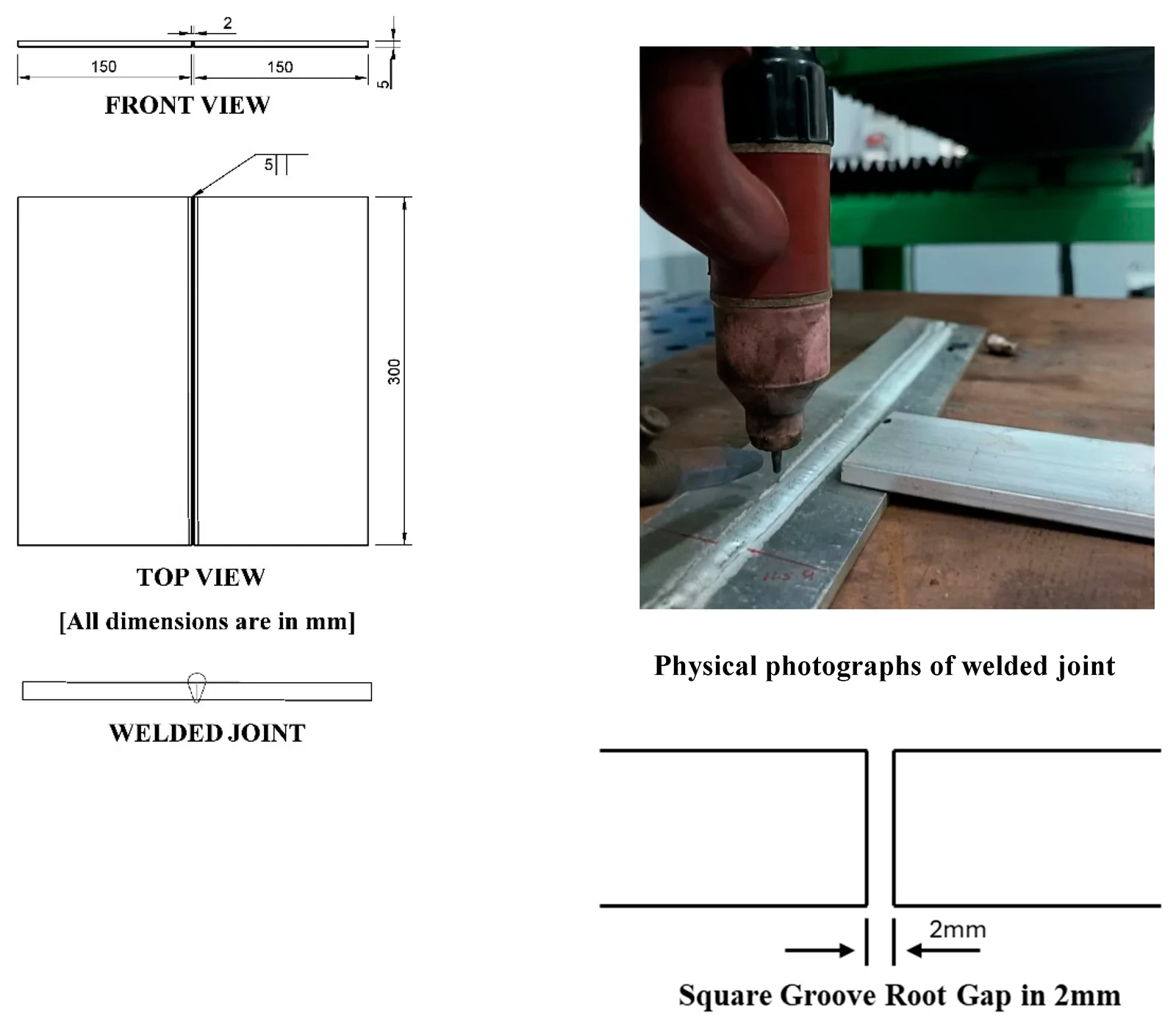
Joint configuration for TIG welding of AA 5083 plates.

**Figure 2:**
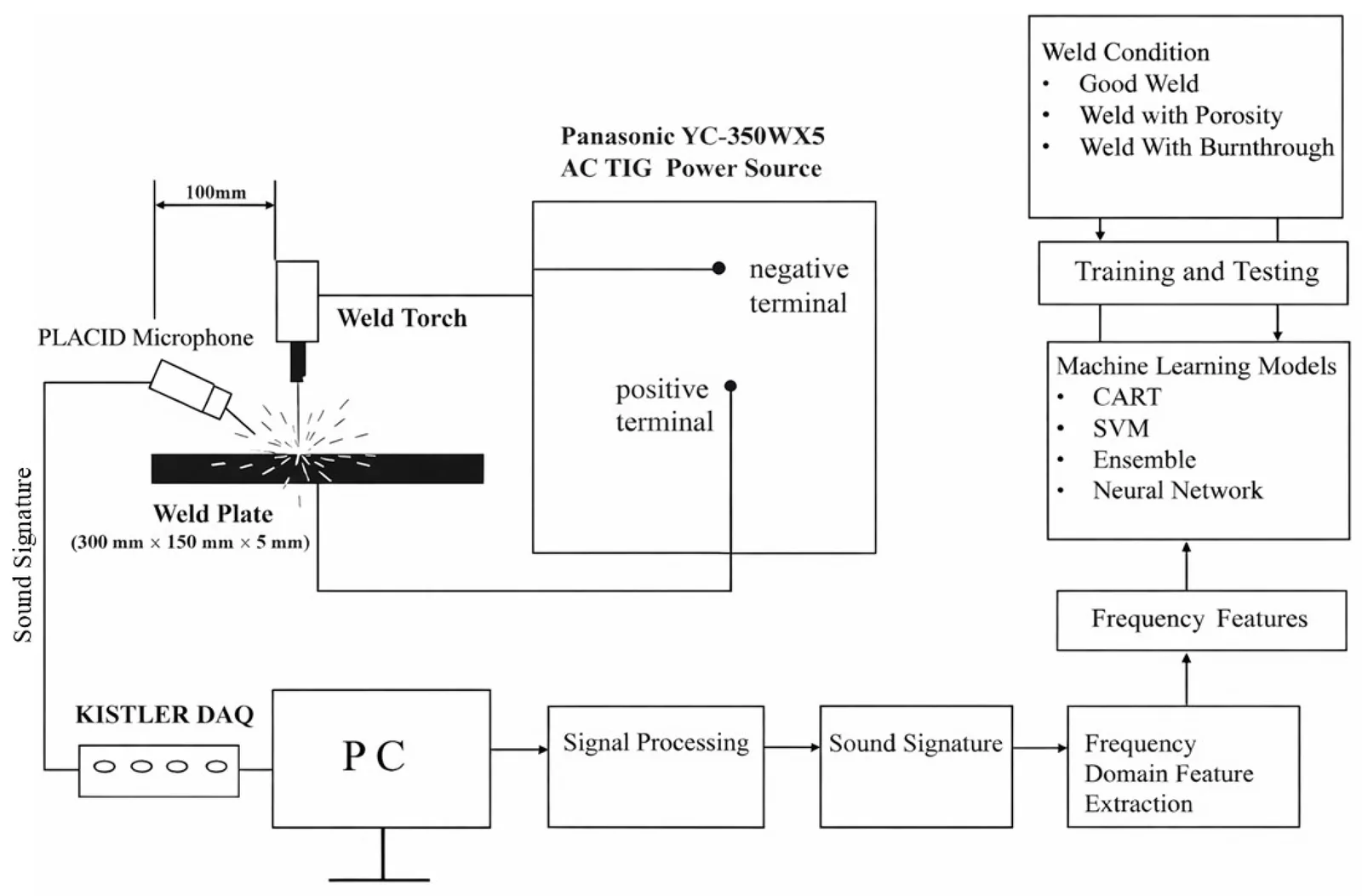
Schematic illustration of the proposed methodology.

**Figure 3:**
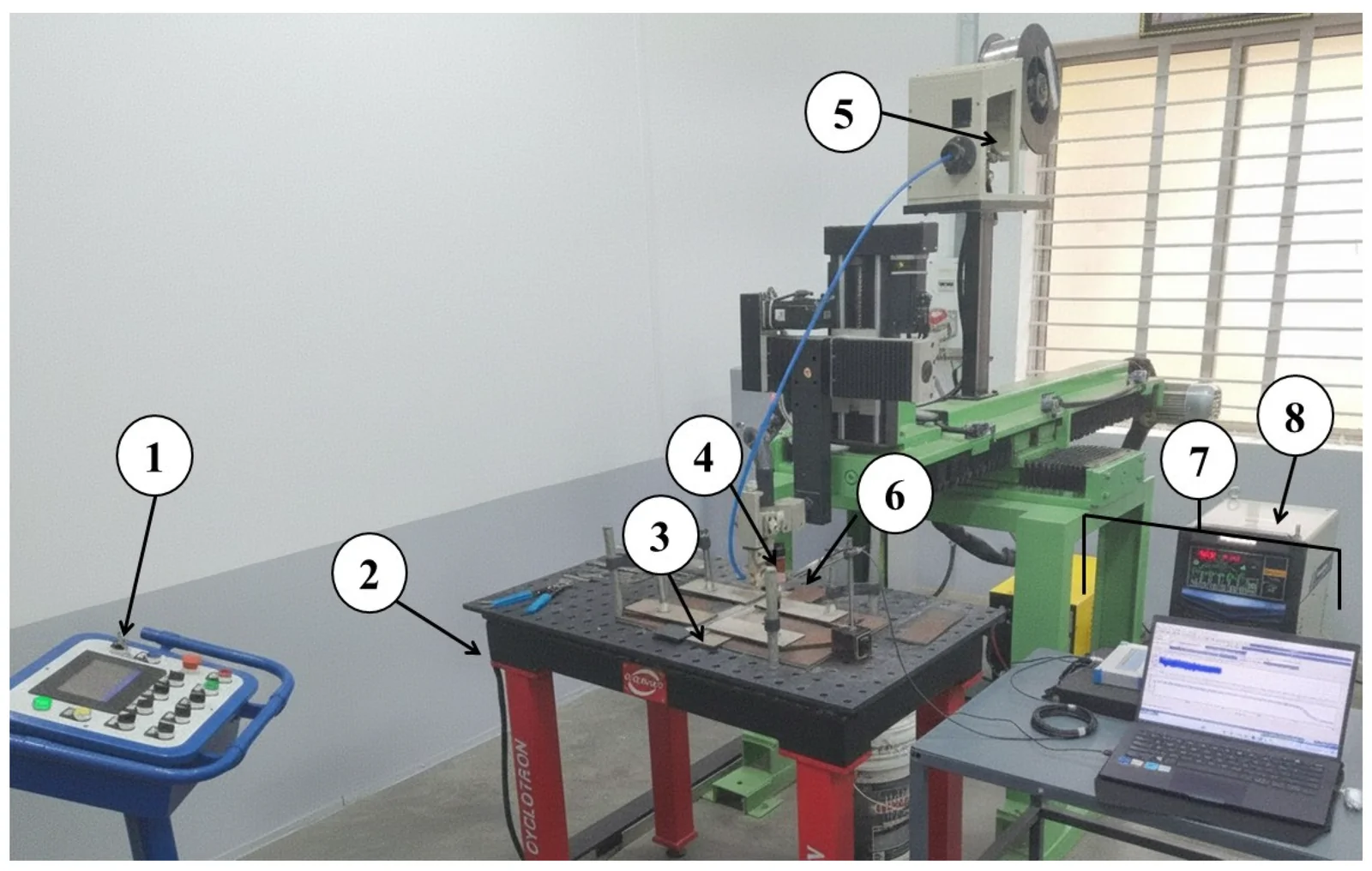
TIG welding experimental setup. Note: (1) controller, (2) table, (3) workpiece, (4) welding torch, (5) wire feed, (6) Kistler data recording setup, (7) PC, and (8) TIG machine.

**Figure 4:**
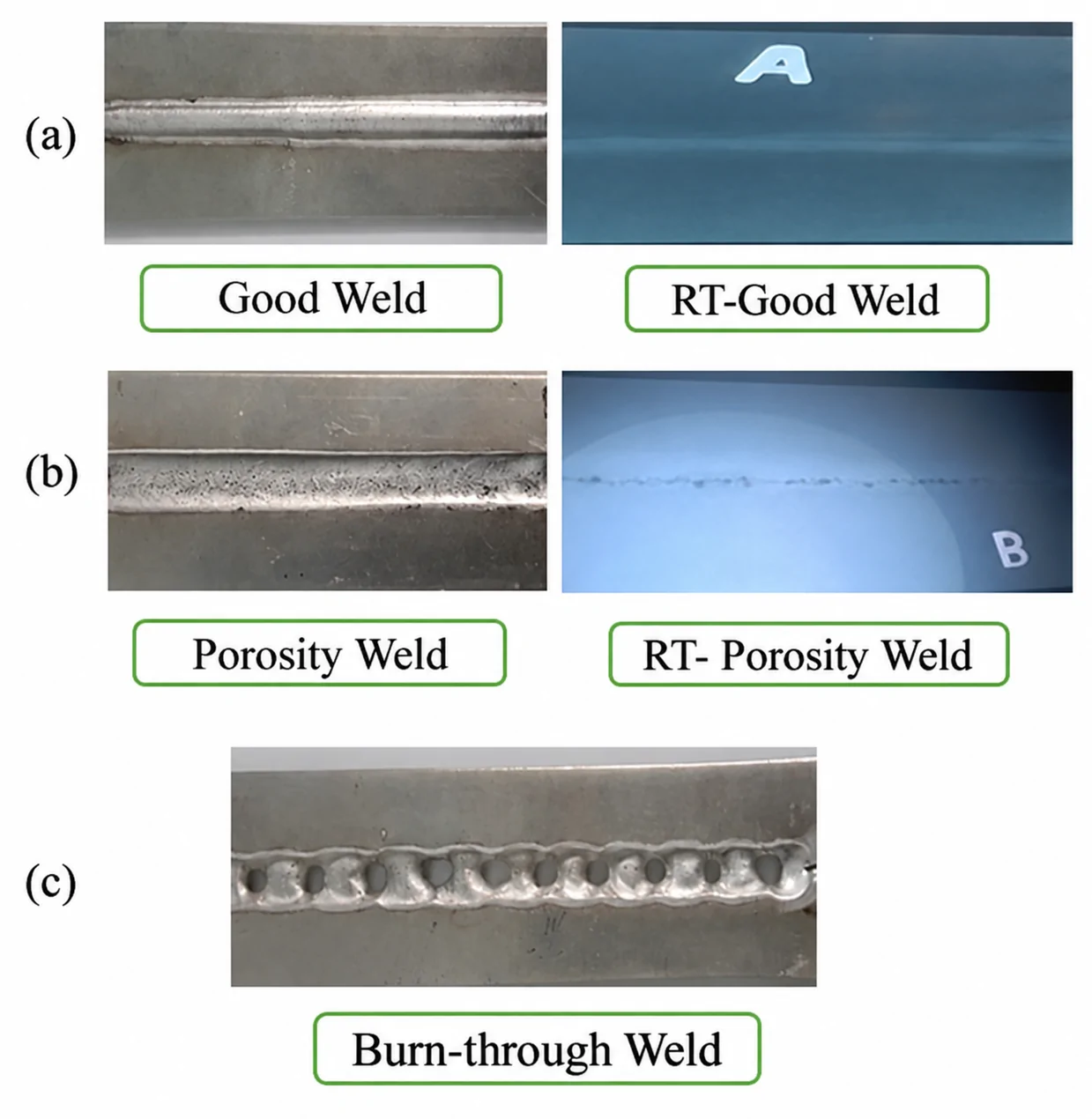
Representative welded joints and corresponding radiographic testing (RT) images for (**a**) a good weld, (**b**) a porosity weld, and (**c**) a burn-through weld.

**Figure 5:**
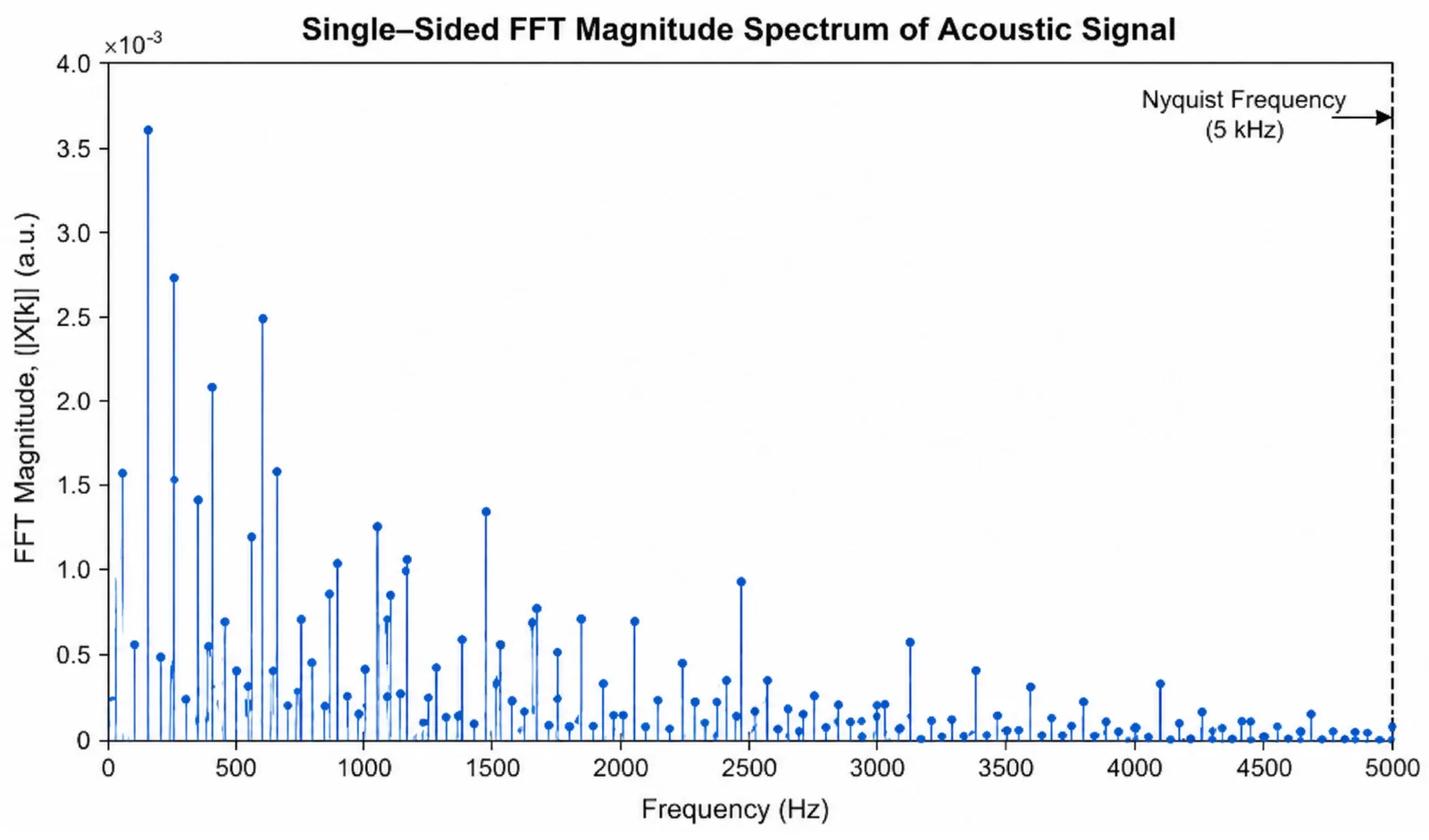
Schematic FFT magnitude spectrum, illustrating the distribution of acoustic energy.

**Figure 6:**
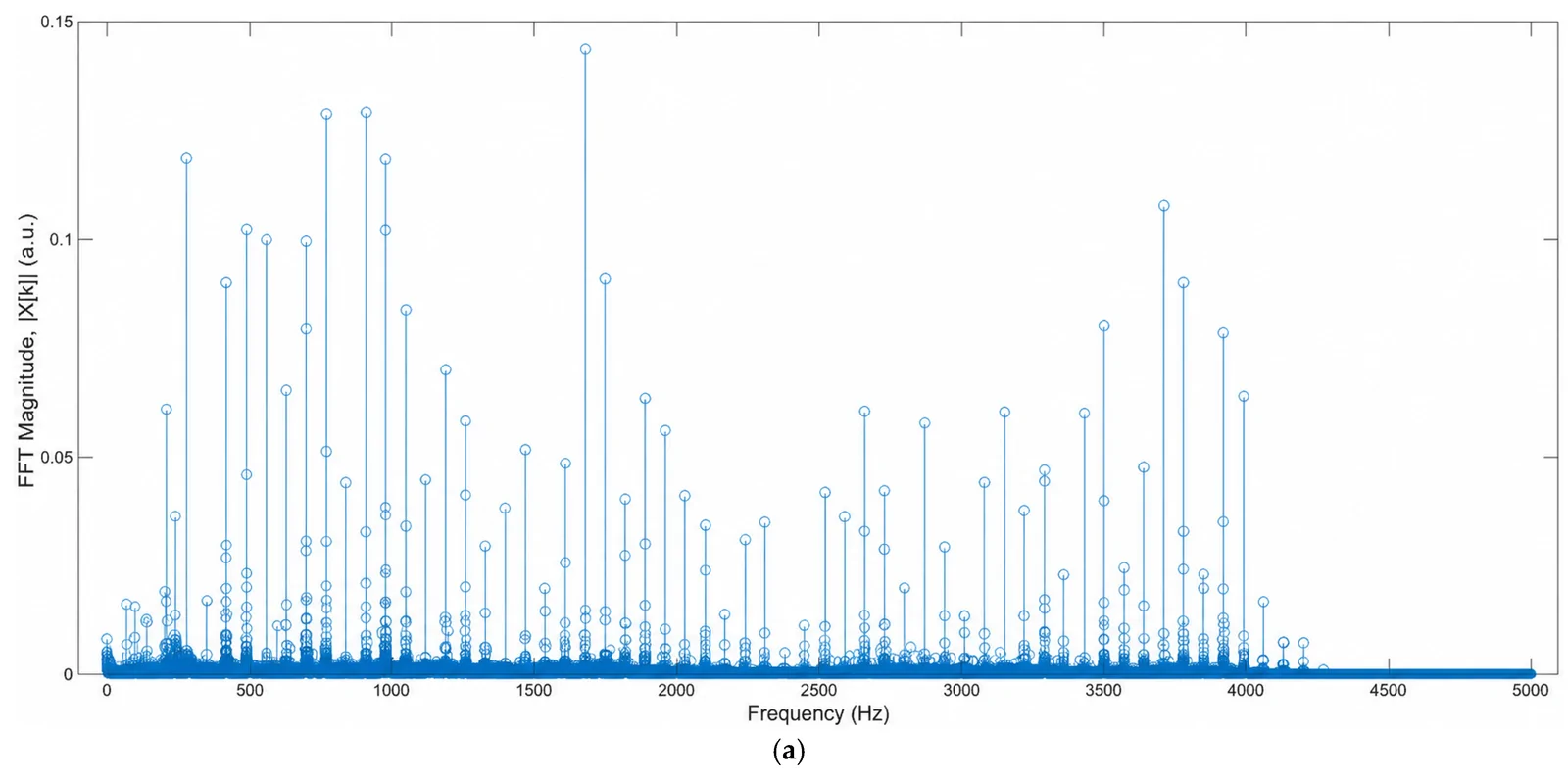

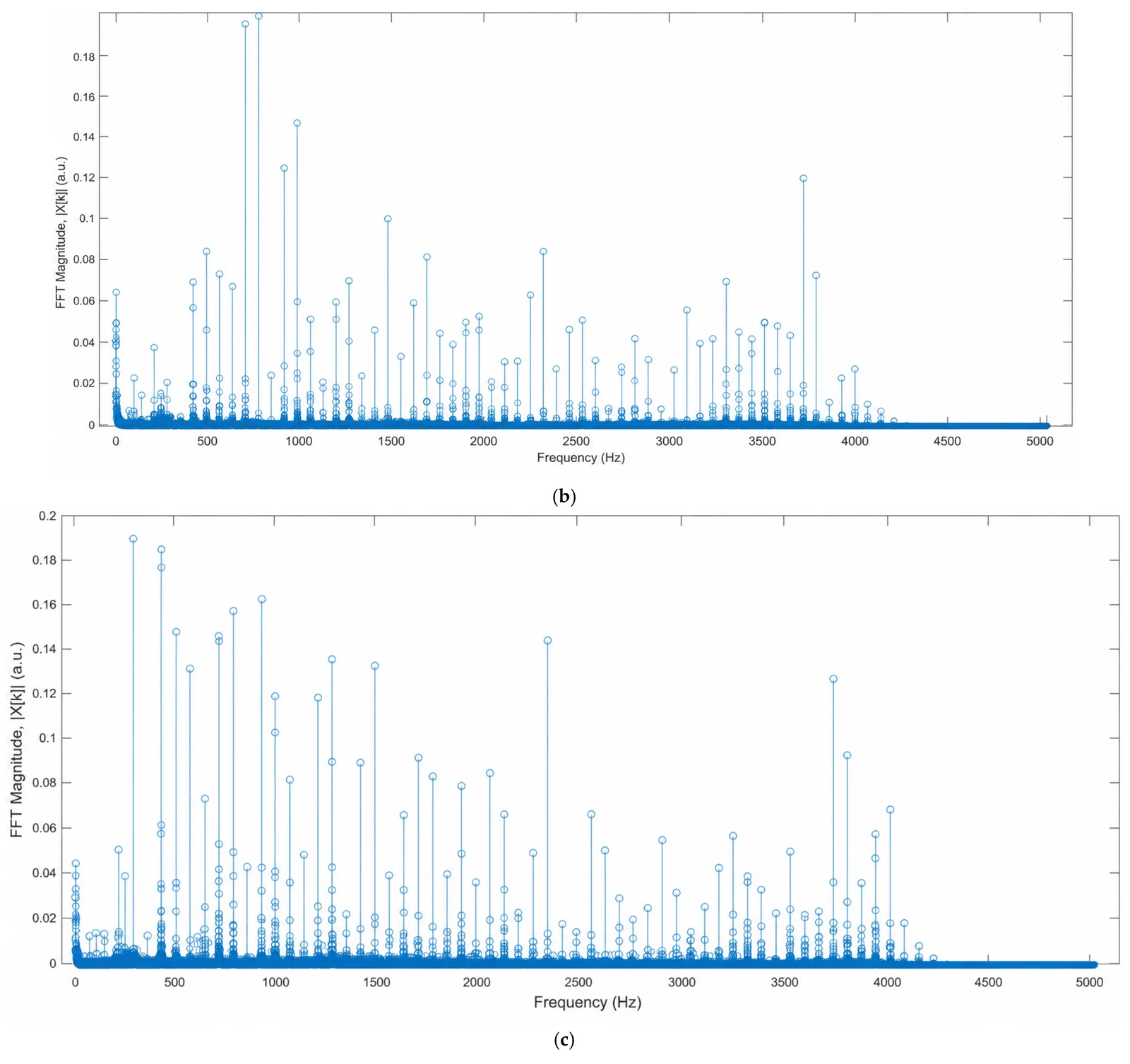
Frequency-domain magnitude spectra of weld signals. (**a**) Good weld; (**b**) porosity defect; (**c**) burn-through defect.

**Figure 7:**
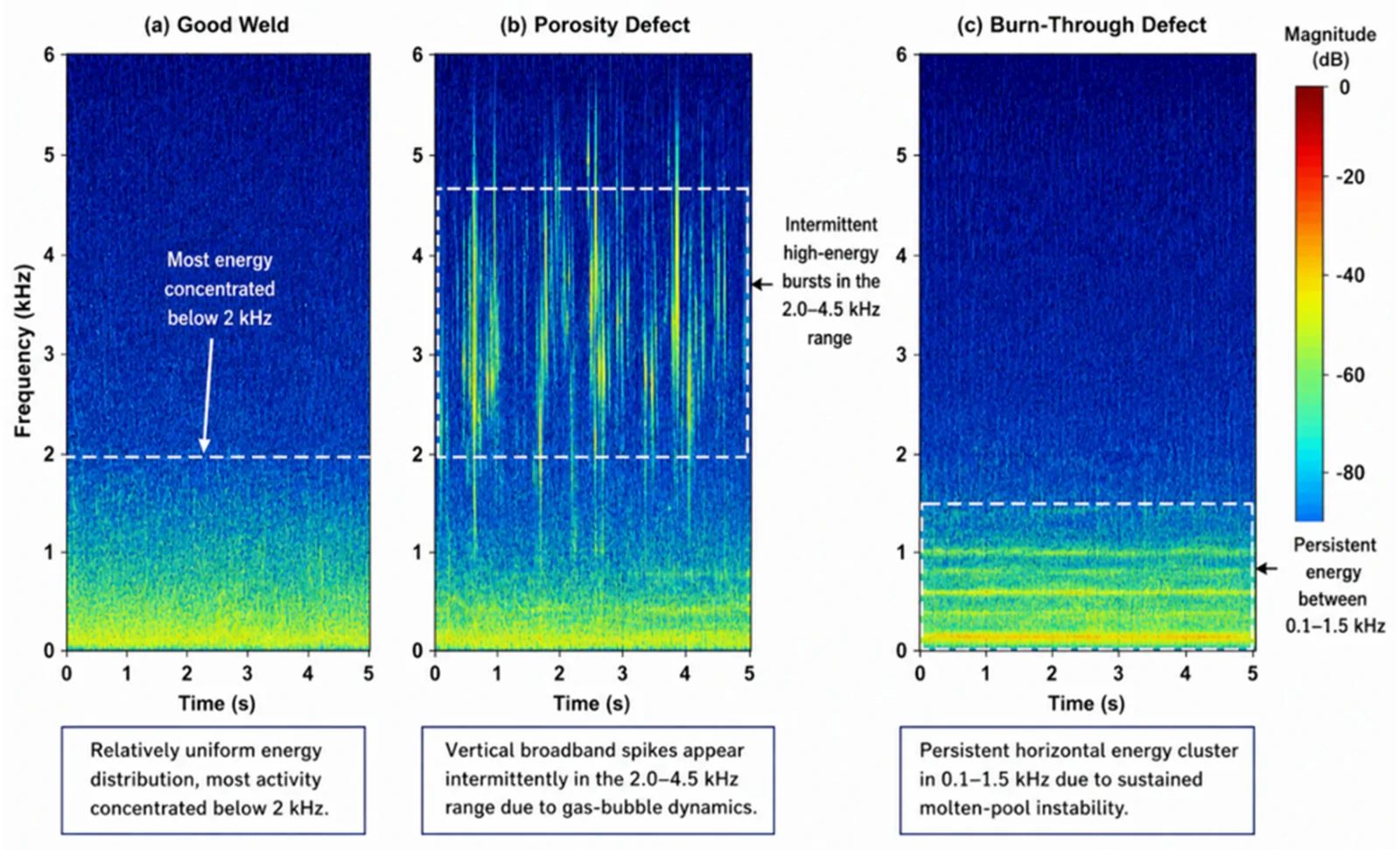
STFT spectrograms of weld signals. (**a**) Good weld. (**b**) Porosity defect. (**c**) Burn-through defect.

**Figure 8:**
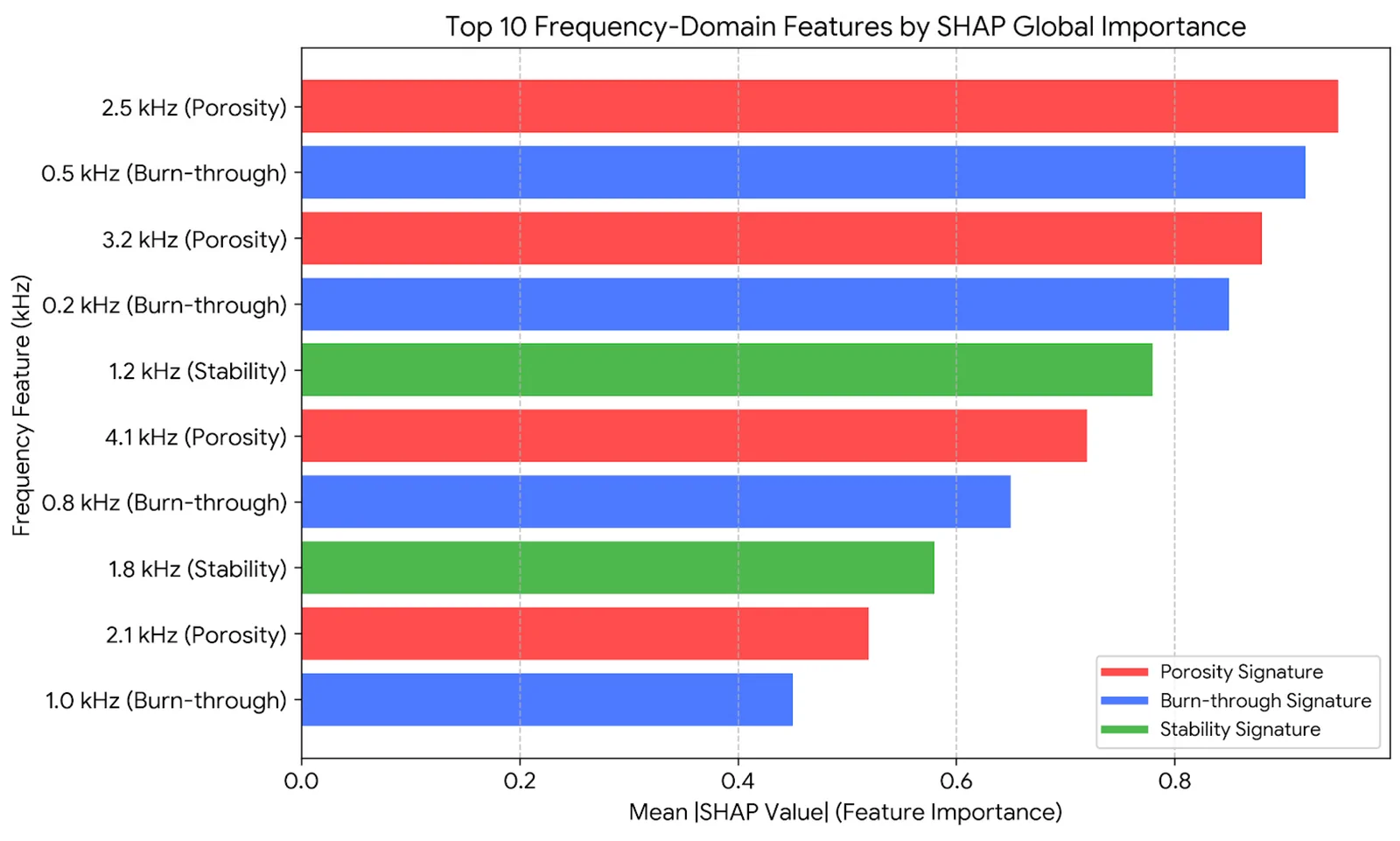
SHAP feature-importance analysis showing the frequency regions contributing to classification of the experimentally investigated weld conditions.

**Figure 9:**
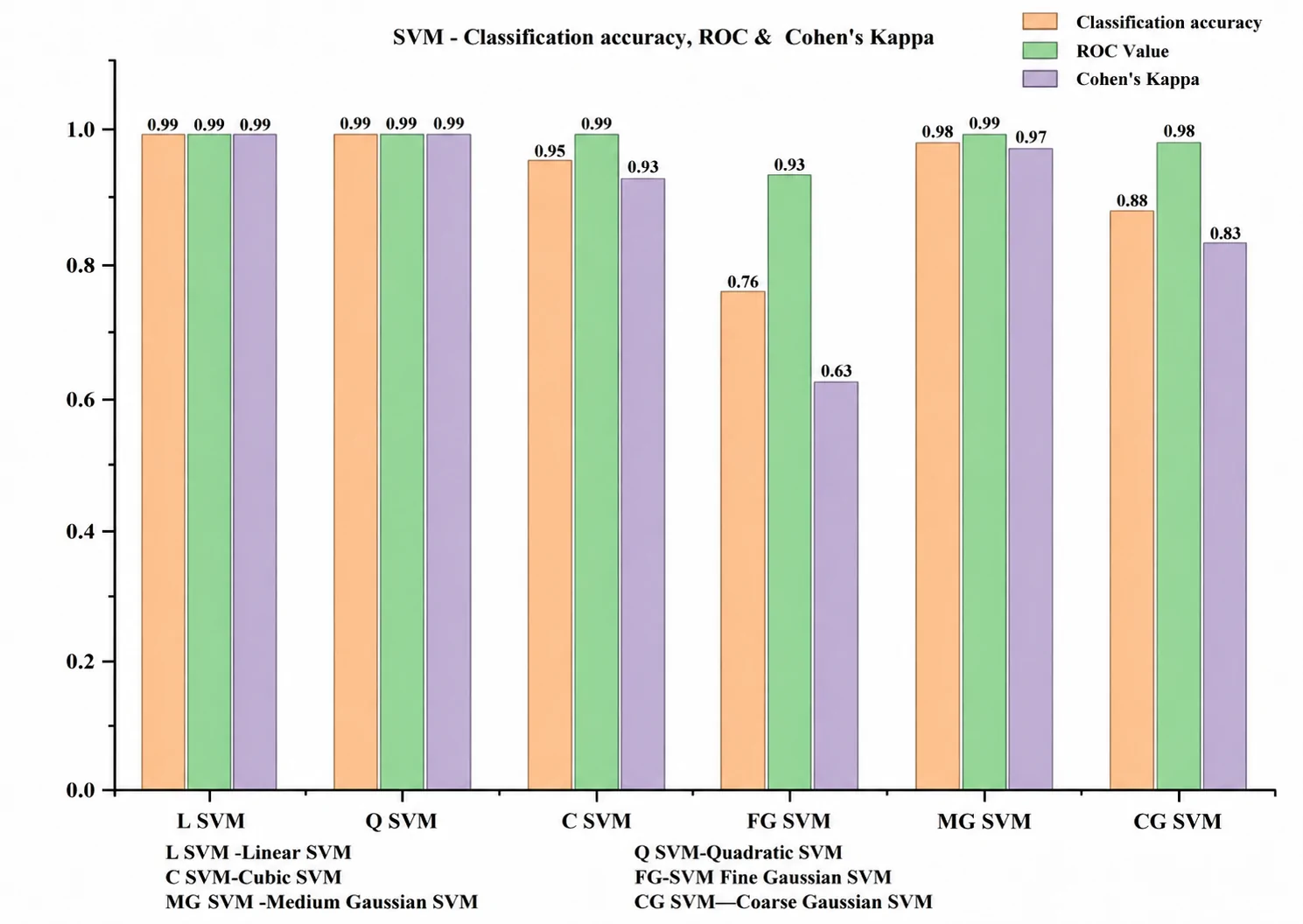
Performance comparison of SVM models.

**Figure 10:**
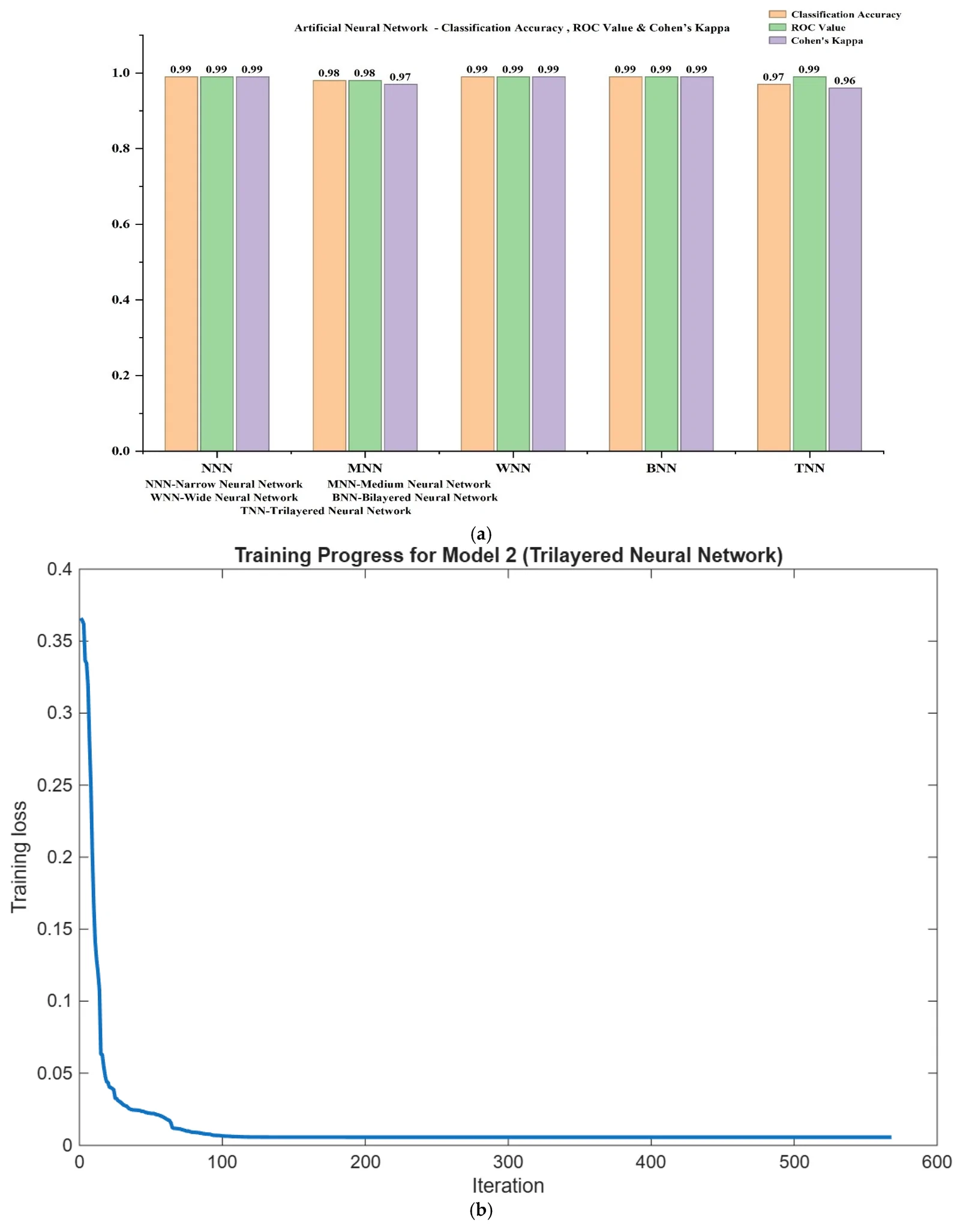
(**a**) Comparative classification performance of the neural network configurations. (**b**) Comparative performance and training convergence of the evaluated ANN configurations.

**Figure 11:**
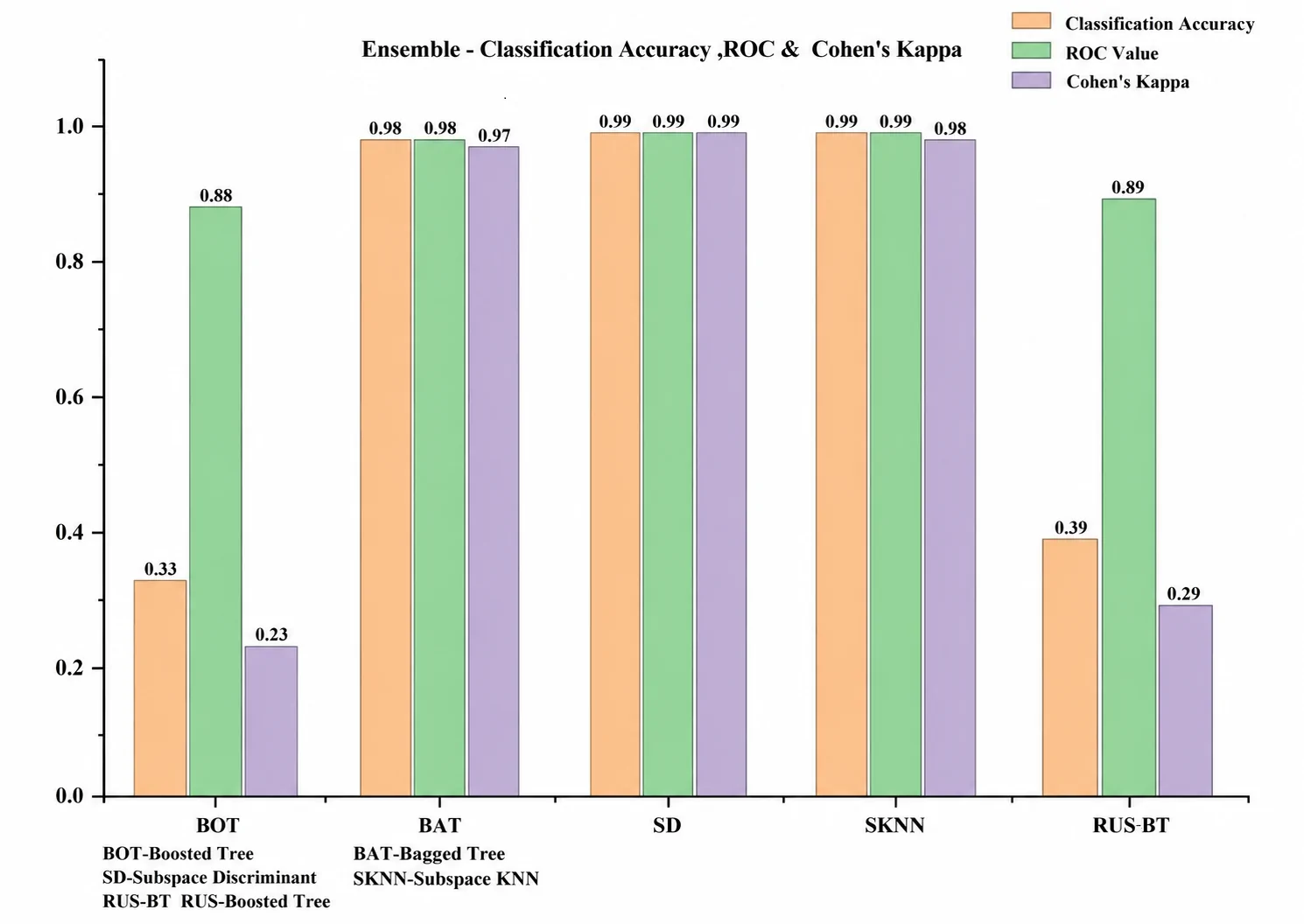
Performance comparison of ensemble models.

**Figure 12:**
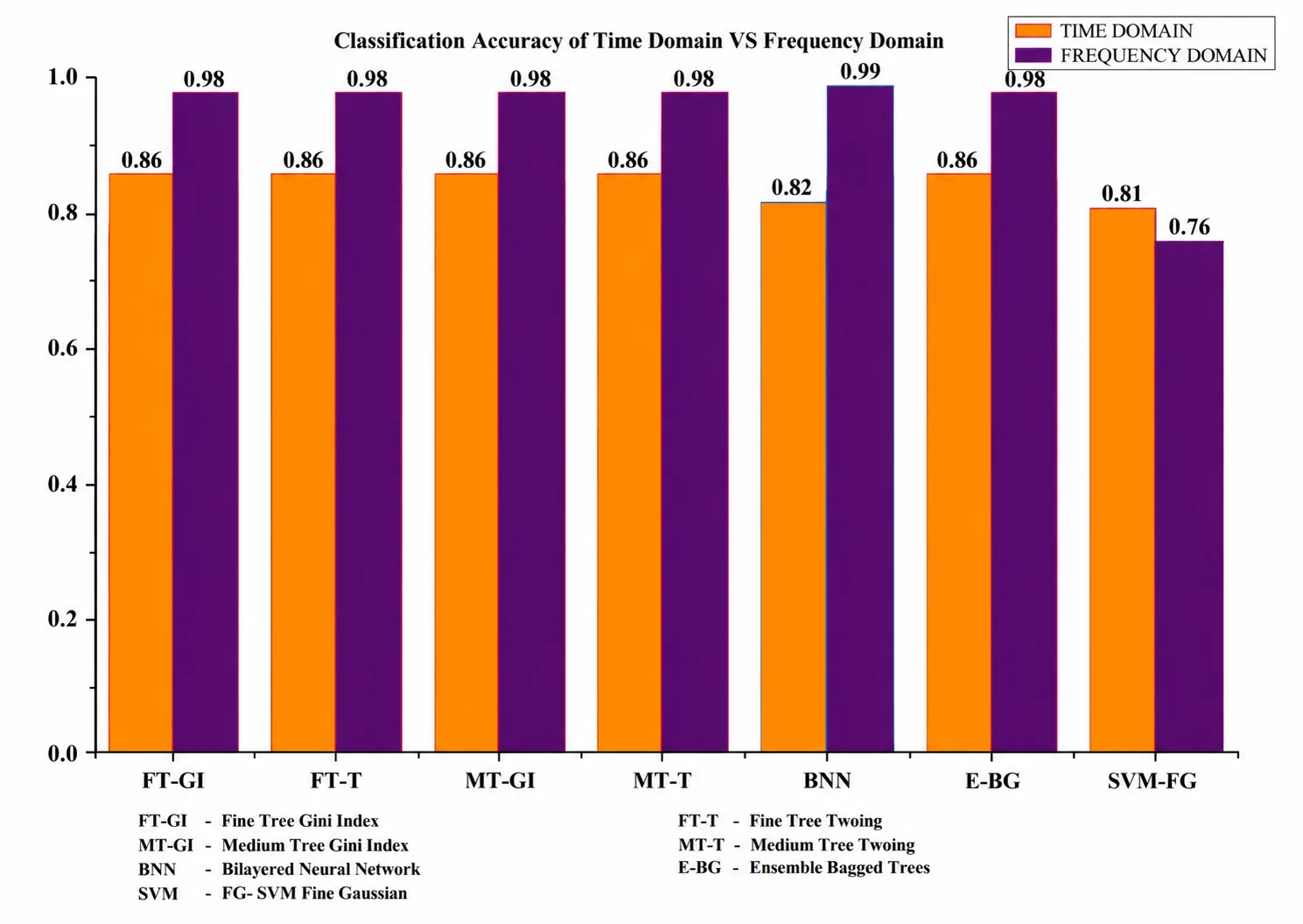
Comparative classification performance of time- and frequency-domain approaches.

**Table 1:** Experimentally measured chemical composition of the AA5083 base alloy.

Element	Fe	Cu	Si	Mn	Cr	Mg	Ti	Zn	Al
Composition (wt.%)	0.250	0.039	0.106	0.505	0.099	4.450	0.018	0.021	94.456

**Table 2:** Chemical composition of the ER5183 filler metal (wt.%).

Element	Fe	Cu	Si	Mn	Cr	Mg	Ti	Zn	Al
Composition (wt.%)	0.4	0.1	0.4	0.5–1.0	0.05–0.25	4.3–5.2	0.15	0.25	92.1–95.2

**Table 3:** Summary of welding parameters.

Parameter	Good Weld	Porosity	Burn-Through
Current (A)	150	200	200
Weld Speed (mm/min)	165	200	200
Wire Speed (mm/min)	450	100	100
Gas Flow Rate (L/min)	10	7	10
Arc Gap (mm)	3	3	3
Root Gap (mm)	2	0	2

**Table 5:** Metrological specifications and uncertainty quantification for acoustic signal acquisition.

Metrological Parameter	Experimental Value/Specification	Impact on Data Integrity and Uncertainty
Sensor Type	PLACID ½ inch prepolarized condenser microphone	High sensitivity and stability for capturing transient weld emissions.
Microphone Placement	100 mm from arc at 45° inclinations	Optimized for high SNR; inclination minimizes grazing incidence and ambient reflections.
Distance Tolerance (±X)	±2 mm	Quantified spatial uncertainty ensures intensity variations remain within \pm 0.2~dB based on the inverse square law.
Angular Tolerance (±Y)	±1°	Maintains consistent capture of directional high-frequency spectral components.
Frequency Response	Flat within 20 Hz–20 kHz range	Ensures magnitude fidelity across the entire 0–5 kHz analyzed Nyquist band.
DAQ Resolution	16-bit (KISTLER system)	Provides a high dynamic range to distinguish low-amplitude defect signatures from the arc noise floor.
Sampling Rate	10 kHz	Sufficient for frequency-domain analysis of TIG arc stability and molten-pool dynamics.

**Table 6:** Summary table for physical mapping.

Weld Condition	Spectral Characteristic	Physical Interpretation
Good Weld	Smooth, dominant low frequencies.	Stable plasma column and uniform pool behavior.
Burn-through	High-magnitude, irregular peaks.	Excessive heat, pool instability, and metal collapse.
Porosity	Localized spikes/peaks.	Gas bubble formation and transient surface bursting.

**Table 7:** Computational comparison of FFT and STFT for acoustic signal analysis.

Metric	(FFT)	STFT
Feature Extraction Time (ms)	~1.2	~4.5
Memory Footprint (KB)	~195	~420
Inference Latency (ms)	~0.8	~1.5

**Table 8:** Hyperparameters of classifiers.

Classifier	Hyperparameters
DT	Split criterion = Gini Index, Max. no. of Split = 100
SVM	Box Constraint Level = 1, Kernel function = Linear SVM, Multi-class method = one vs. one
ANN	Number of fully connected layer = 1, First Layer Size = 10, Activation = ReLU, Iteration Limit = 1000, Regularization Strength (Lambda) = 0, standardize Data = Yes
Ensemble	Ensemble method = Subspace Discriminant, Learner Type = Nearest Neighbors, No. of Learners = 30, Subspace Dimension = 3

**Table 11:** Confusion matrix of the Quadratic SVM classifier trained using FFT-derived frequency-domain acoustic features.

		B	G	P
Actual Class	B	60	0	0
	G	0	59	0
	P	1	0	59
**Evaluated Parameters**				
Classification Accuracy	0.99		Precision	0.99
ROC Value	0.99		F1-Score	0.99
Kappa Statistics	0.99		Specificity	0.99
Sensitivity	0.99		Prevalence	0.33
False Discovery Rate (FDR)	0.54		Miss rate	0.55

(B: Burn-through; G: good weld; P: porosity.)

**Table 12:** Confusion matrix of the single-hidden-layer ANN (10 neurons) classifier trained with sound features.

		B	G	P
Actual Class	B	60	0	0
	G	0	59	0
	P	1	0	59
**Evaluated Parameters**				
Classification Accuracy	0.99		Precision	0.99
ROC Value	0.99		F1-Score	0.99
Kappa Statistics	0.99		Specificity	0.99
Sensitivity	0.99		Prevalence	0.33
False Discovery Rate (FDR)	0.54		Miss rate	0.56

(B: Burn-through; G: good weld; P: porosity.)

**Table 13:** Confusion matrix of the selected ANN classifier.

Validation Confusion Matrix for Model 2 (Tri-Layered Neural Network)				
		B	G	P
Actual Class	B	42	0	0
	G	0	42	0
	P	1	0	41

(B: Burn-through; G: good weld; P: porosity.)

**Table 14:** Confusion matrix of the ensemble subspace discriminant classifier trained with sound features.

		B	G	P
Actual Class	B	60	0	0
	G	0	59	0
	P	1	0	59
**Evaluated Parameters**				
Classification Accuracy	0.99		Precision	0.99
ROC Value	0.99		F1-Score	0.99
Kappa Statistics	0.99		Specificity	0.99
Sensitivity	0.99		Prevalence	0.33
False Discovery Rate (FDR)	0.54		Miss rate	0.56

(B: Burn-through; G: good weld; P: porosity.)

**Figure A1:**
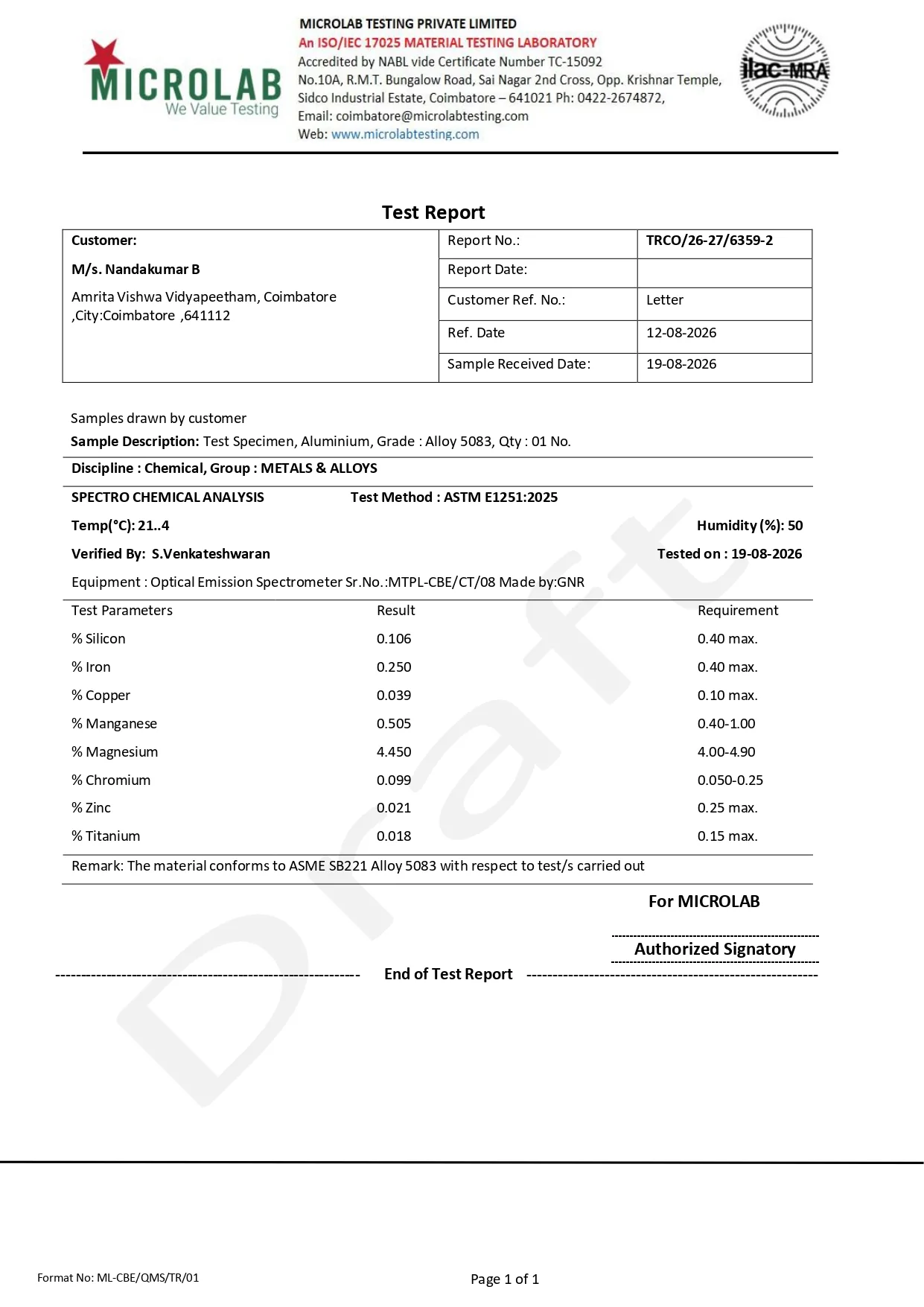
Chemical Composition of AA5083.

## Data Availability

The raw data supporting the conclusions of this article will be made available by the authors on request.

## Appendix A

### Appendix A.1. Laboratory Report for Chemical Composition of AA5083

The laboratory report provides the original spectrochemical analysis of the AA5083 test specimen. The report identifies the specimen as Aluminum, Grade Alloy 5083, and states that spectrochemical analysis was performed according to ASTM E1251:2025 using an optical emission spectrometer. The reported results are Si 0.106%, Fe 0.250%, Cu 0.039%, Mn 0.505%, Mg 4.450%, Cr 0.099%, Zn 0.021%, Ti 0.018%, and Al 94.456% (remainder). The report remarks that the material conforms to ASME SB221 Alloy 5083 [38] with respect to the tests carried out.

### Appendix A.2. Worked Example for Calculation of Evaluation Parameters

This appendix demonstrates, using the Decision Tree confusion matrix, how the evaluation parameters reported in Section 3.4.1 are calculated. The same definitions and calculation procedure were applied to the SVM, ANN, and ensemble classifiers.

**Table A1:** Decision Tree confusion matrix used for the worked example.

Actual/Predicted	B	G	P
B	59	1	0
G	0	59	0
P	2	0	58

Here, B = burn-through, G = good weld, and P = porosity. The matrix contains 180 observations. The diagonal values (59, 59, and 58) are correctly classified samples, while the off-diagonal values are classification errors.

**1. Classification accuracy**
  Accuracy = (59 + 59 + 58)/180 = 176/180 = 0.9778 ≈ 0.98.
**2. Precision**
  Precision = TP/(TP + FP). For B: 59/(59 + 2) = 0.9672. For G: 59/(59 + 1) = 0.9833. For P: 58/(58 + 0) = 1.0000. The macro-average precision is approximately 0.9835.
**3. Sensitivity (Recall)**
  Sensitivity = TP/(TP + FN). For B: 59/(59 + 1) = 0.9833. For G: 59/(59 + 0) = 1.0000. For P: 58/(58 + 2) = 0.9667. The macro-average sensitivity is approximately 0.9833.
**4. Specificity**
  Specificity = TN/(TN + FP). For B: 117/(117 + 2) = 0.9832. For G: 119/(119 + 1) = 0.9917. For P: 119/(119 + 0) = 1.0000. The macro-average specificity is approximately 0.9916.
**5. F1-score**
  F1 = 2(Precision × Recall)/(Precision + Recall). The class-wise F1 values are approximately 0.9752 for B, 0.9916 for G, and 0.9831 for P, giving a macro-average of approximately 0.9833.
**6. Cohen’s Kappa**
  Observed agreement, Po = 176/180 = 0.9778. Expected agreement, Pe = [(60 × 61) + (59 × 60) + (61 × 59)]/180^2^ ≈ 0.3335. Therefore, Kappa = (Po−Pe)/(1−Pe) ≈ 0.9666 ≈ 0.97.
**7. False Discovery Rate (FDR):**
  FDR = FP/(TP + FP). Across the three classes, FP = 2 + 1 + 0 = 3. Therefore, aggregate FDR = 3/180 = 0.0167 ≈ 1.67%.
**8. Miss rate**
  Miss rate = FN/(TP + FN). Across the three classes, FN = 1 + 0 + 2 = 3. Therefore, aggregate miss rate = 3/180 = 0.0167 ≈ 1.67%.
**9. Prevalence**
  Prevalence is the proportion of actual samples belonging to a class. For B: 60/180 = 0.33; for G: 59/180 ≈ 0.33; for P: 61/180 ≈ 0.34. The approximately balanced dataset therefore gives a prevalence close to 0.33 per class.
  The worked example is provided once to make the calculation procedure transparent. The same formulas were used for the confusion matrices of the SVM, ANN, and ensemble models; therefore, repeating the full calculation for every model is unnecessary.
  Final editorial check: use the final Decision Tree confusion matrix table number consistently in Section 3.4.1 and Appendix A.2 after the manuscript’s table numbering has been finalized.
  ********************
